# Predicting Transiently Expressed Protein Yields: Comparison of Transfection Methods in CHO and HEK293

**DOI:** 10.3390/pharmaceutics14091949

**Published:** 2022-09-14

**Authors:** Ly Porosk, Jekaterina Nebogatova, Heleri Heike Härk, Birgit Vunk, Piret Arukuusk, Urve Toots, Mart Ustav, Ülo Langel, Kaido Kurrikoff

**Affiliations:** 1 Institute of Technology, University of Tartu, Nooruse 1, 50411 Tartu, Estonia; 2 Icosagen Cell Factory, Eerika tee 1, Õssu, 61713 Tartu, Estonia

**Keywords:** transient transfection, protein expression, transfection, cell-penetrating peptides, protein production, CHO, HEK293, mAb production

## Abstract

Therapeutic proteins are currently at the apex of innovation in pharmaceutical medicine. However, their industrial production is technically challenging and improved methods for transient transfection of mammalian cell cultures are necessary. We aimed to find a fast, microliter-scale transfection assay that allows the prediction of protein expression in the transient production settings. We used an array of lipid, polymeric and cell-penetrating peptide transfection reagents, and compared their performance in various high throughput transfection assays to their performance in protein (antibody) expression in professional protein-producer cell lines. First, we show that some of the most frequently used microliter-scale transfection efficacy assays fail to predict performance in the protein production in milliliter and liter scale settings. We found that CHO suspension culture post-transfection EGFP(+) population and SEAP quantitation correlate with large-scale protein production, whereas the adhesion culture assays and transfection of pLuc are non-predictive. Second, we demonstrated that cell-penetrating peptide-based transfection achieves significantly higher protein yields compared to PEI and lipoplex methods in both CHO and HEK293 producer cell lines. In this work we demonstrate a CPP-based transient protein expression approach that significantly outperformed the current industry standard workhorse method of PEI.

## 1. Introduction

Therapeutic proteins represent a huge and growing field in pharmaceutical medicine: half of the global top 10 revenue drugs sold in 2021 were either humanized monoclonal antibodies (mAb) or other protein-based biologicals [1]. Therapeutic intervention using mAb has proven to be very successful, especially in cancer and inflammatory diseases. mAb allow treatment with improved efficiency and reduced side effects at a magnitude that has not been previously achievable with chemically synthesized small-molecule drugs [2]. 

Ongoing research efforts are critical for the development of efficient methods that offer robust transgene expression and thus meet the growing demand for recombinant proteins. The industrial production of protein drugs is considerably more challenging than synthesizing low molecular weight compounds chemically and thus the improvement of current cell culture manipulation methods has an immense importance [3]. Protein drugs are produced in cultivated mammalian cells to ensure their proper folding, assembly, and post-translational modifications. For efficient protein production with high yields several challenges have to be overcome. One of these is the process of transfection where a plasmid DNA (pDNA) expression vector is introduced into the cells to use the cells’ protein biosynthesis machinery [4]. Several methods have been developed for performing transient chemical transfection in mammalian cells, and all of these are derived from the methods of lipofection [5], polyethylenimine [6], and CPPs [7]. An overview of the state of the art of these non-viral methods can be found in recent reviews [8,9,10].

Transient transfection was introduced for mass use (with scale-up compatibility) in 1995 through the polyethylenimine (PEI) method [6]. Since then, transient transfection has been the first choice for rapidly obtaining recombinant proteins in industrial settings without the need to spend time and resources on stable cell line development [4]. The technology has leapt forward with the use of cell engineering and culturing advancements—protein yields that used to be in the range of mg/L 20 years ago have now increased to an impressive 1–2 g/L in CHO and HEK293 producer cell lines [3]. 

Nevertheless, efficient transfection and subsequent protein expression cannot be achieved by a universal method, and the outcome of transfection and protein expression is influenced by many aspects, such as the cell lines utilized, the culturing conditions, etc. [3]. Among a plethora of different chemical transfection reagents available to choose from, the majority have been developed for the microliter scale. The aim of this work is to assess the predictive power of microliter-scale transfection reporter assays in large-scale protein expression to provide tools to optimize transient transfection prior to launching the liter-scale production process.

## 2. Results and Discussion

### 2.1. Classically Used Transfection Efficacy Assays Fail to Predict Protein Production in Industrial Settings

Polyplex (PEI) and lipoplex-based approaches are the most frequently used transient transfection methods [4]. The transfection efficacy can be assessed by the quantitation of the expressed reporter. The total luciferase amount is often measured from the cell lysate in an adherent cell culture between 24 h and 48 h post-transfection. Luciferase reporter assays are sensitive, fast, require simple equipment, and are widely available. Since the desired outcome in the industry is maximizing the yields of protein-of-interest, it is therefore reasonable to focus on the quantitation of total reporter protein. 

**Figure 1:**
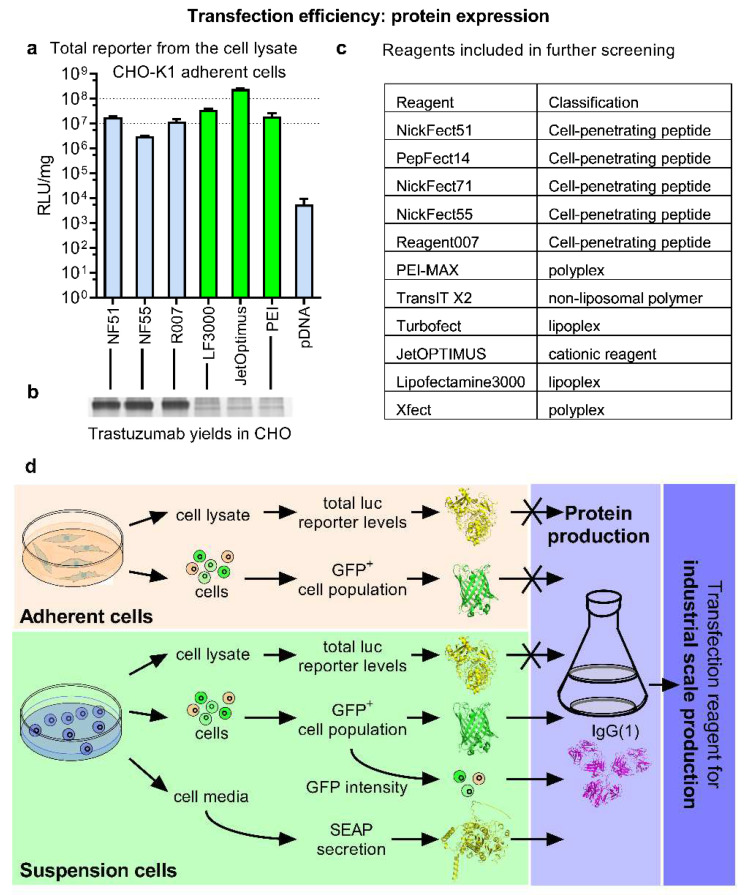
The total expressed reporter levels in adherent cells do not correlate with therapeutic protein production efficacy. (**a**) Transfection efficacy is assessed by quantitation of luminescence from cell lysate 24 h post-transfection of CHO-K1 adherent cells with pLuc. The top performers are highlighted in green. N = 5. (**b**) The production of Trastuzumab mAb in CHO 1H7 suspension cell culture in serum-free defined media 7 days after the transient transfection of pDNA. The complexes with PEI were formed at N/P ratio 20, and the complexes with CPPs were formed at CPP/pDNA charge ratio 2.5:1. They were assessed by SDS-PAAG 10%, with Coomassie blue staining. (**c**) List of reagents included in the further study. (**d**) A scheme depicting general workflow.

**Figure 2:**
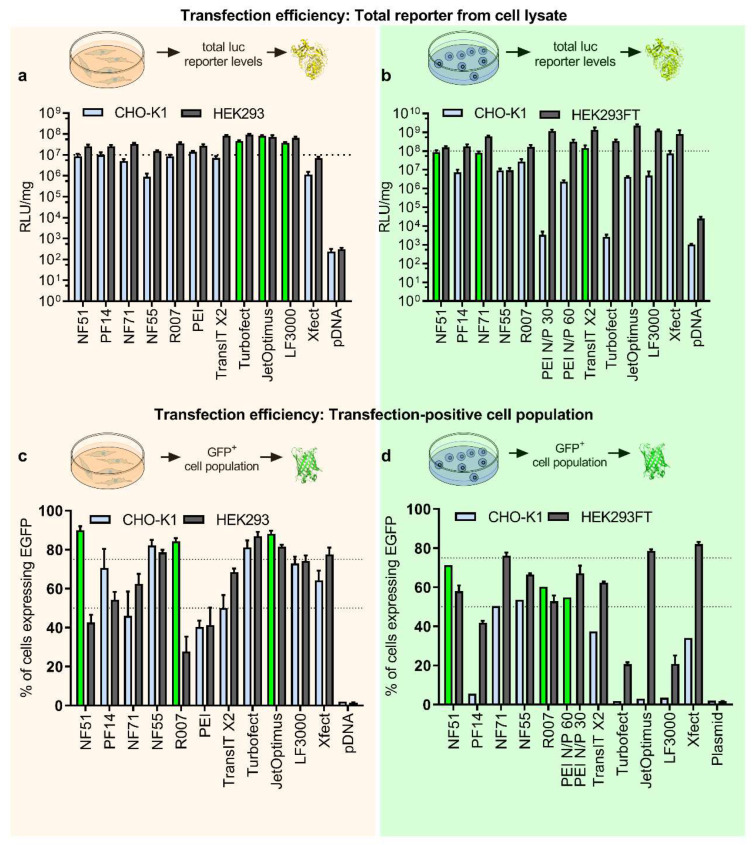
Suspension culturing improves the predictability of protein production. (**a**) Adherent cells were transfected with 0.2 µg of pLuc per 96-well plate well. CPP/pDNA complexes were formed at CR3:1, and PEI complexes were formed at N/P20. The total reporter levels were determined 24 h post-transfection. N = 10 for treatment groups. (**b**) The suspension cells were transfected with 0.1 µg of pLuc per 96-well plate well. CPP/pDNA complexes were formed at CR2:1. Total reporter levels were determined 48 h post-transfection. N = 10 for treatment groups. (**c**) Adherent cells were transfected in serum-free media with 0.5 µg of green fluorescent protein encoding plasmid (pGFP) per 24-well plate well. 4 h post-transfection media was changed to serum-containing media. CPP/pDNA complexes were formed at CR3:1, PEI N/P20. Analysis was performed 24 h post-transfection. N ≥ 5. (**d**) Suspension cells were transfected in serum-free media with 0.75 µg of pGFP per 24-well plate well. CPP/pDNA complexes formed at CR2:1. For PEI N/P20 was used in case of the HEK293FT cells and N/P60 in case of CHO-K1 cells. 4 h post-transfection 500 µL of fresh media was added to each well. 48 h post-transfection the cells were analyzed by flow cytometry. N ≥ 6 for treatment groups.

The pDNA transfection efficacy (Figure 1a,b) of a small set of well-known lipoplex, polyplex, as well as cell-penetrating peptide (CPP)-based transfection methods were compared (Figure 1c) in CHO cells, which is the primary expression platform [3,11]. We normalized the relative luminescence units (RLU) with total protein, as this does not change the tendencies between the reagents (Figure S1), but allows the comparison of RLUs from different experiments. Since each transfection reagent has its own instructions for use, leaving room for optimization in many cases, assay conditions were selected by optimizing the reagent amount (Figure S2a), N/P ratio (Figure S2b), charge ratio (Figure S2c), media change and pDNA dose (Figure S3a–c) to maximize the transfection output. Generally, a high-protein expression was observed with all the methods, although lipoplex and polyplex proved to be the most effective (Figure 2a). CPP-mediated transfection, albeit efficient, did not quite reach the levels shown by jetOptimus or Lipofectamine. When taking into account the mean values, CPPs also performed slightly less efficiently than PEI-Max.

As a next step we investigated how these reporter performances translated into the industrial protein expression settings. The production of an example protein, therapeutic mAb Trastuzumab, was used to achieve this with the QMCF protein expression technology [12] in CHO suspension cells over a one-week production cycle. The QMCF includes Epstein-Barr Virus nuclear antigen 1 (EBNA-1) and mouse polyomavirus Large T antigen (PyLT) elements in the producer cells and plasmid expression vectors with the aim of enhancing the retention and replication of the protein expression vector in the cells and allowing significant extension of the “transient” period in the protein production. Other protein expression methods with similar mechanisms are also available [3]. We hypothesized that all the transfection methods were able to produce reasonable yields of mAb, especially the top performers from Figure 1a as these mediated high reporter protein expression from the same cell line in a short time frame. Unexpectedly, significant differences from the assumed output were observed—the CPP methods NF55 [13], NF51 [14], as well as the Reagent007 all significantly outperformed both the lipoplex and polyplex approaches in produced protein levels (Figure 2b). Considering this, we conclude that the fast and easy luciferase reporter lab assay fails to predict industrial protein production performance (Figure 2d). 

Considering that clinically relevant recombinant protein production in mammalian cells is, by its nature, technically challenging, the whole field benefits from being able to predict large-scale performance without weeks of production in cell cultures [15]. The availability of fast lab-scale screening methods would contribute to the development of improved transfection methods for protein-based drug production, and protein production in general.

To investigate the transfection efficacies and possibilities for protein production further, we expanded the selection of transfection methods and included CPPs NF71 [16], PF14 [17], and other known examples of polymers and liposomes (Figure 1c). Furthermore, to explore if the abovementioned discrepancy was specific to CHO, we included another cell line, HEK293. HEK293 is a well-established producer cell line because of its ease of use for both cell growth and transfection, as well as its excellent protein yields. HEK293 is frequently utilized as the expression system to produce recombinant proteins and viruses for gene therapies [4]. Again, to be able to compare different reagents, optimal conditions for reagents in HEK cells were determined to maximize the transfection result (Figures S2a,b and S3b,c). We completed luciferase expression with the extended set of transfection methods and observed that the output efficacies were in line with those observed with the initial smaller set in Figure 1a. The top performers were liposomal and polymeric approaches with statistically significantly higher values (Figure 2a, light blue bars, top three are highlighted in green). For the HEK293 cell line, the differences between the methods were smaller, but liposomal and PEI-based approaches were still the top performers (Figure 2a).

An important difference between small-scale and the industrial cell factories is the use of suspension cultures. With the use of suspension cells, much higher cell densities can be achieved than possible with adherent cell cultures. Therefore, as a next step, we assessed the transfection efficacies in suspension culture by using a total luciferase quantitation assay. The transfection time and volume, presence of serum in the media, transfection reagent dose, pDNA dose, and N/P ratio were optimized to maximize the expressed protein levels (Figures S4a,b and S5a–d). 

In the suspension culture, the contrast between high and low performers was further pronounced (Figure 2a vs. Figure 2b). Notably, the top performers in CHO suspension culture were markedly different from what were observed in adherent conditions, and included two peptide-based methods (NF51 and NF71) and one polyplex-based (TransIT) method (Figure 2b, highlighted in green). Importantly, when comparing the high and low performers in the suspension culture to the initial set of methods included in mAb production (Figure 1b), the predictive value of the suspension culture transfection was higher than that observed in adherent cell culture. One of the most efficient protein producers—NF51—was now among the top three transfection methods, and none of the nonperformers in mAb production were among the best transfection methods in CHO suspension culture (Figure 2b vs. Figure 1b).

### 2.2. The Transfected Cell Population Is an Important Protein Expression Efficacy Predictor

Although the amount of the total expressed protein (Figure 2a,b) could be an intuitive way for estimating transfection performance, long-term protein production is also dependent on another important aspect—a successful division of transfected cells. Each transfection-positive cell clone gives rise to approximately 100 to 500 protein-producing offspring, depending on the cell line and cultivation conditions [18]. For example, a cell that is successfully transfected (estimated at an early time point, such as in Figure 2), but is unable to undergo cell division and produce a high number of daughter cells (which will only become apparent after several days post-transfection, such as in Figure 1b) is probably not contributing to high levels of expressed protein yields. To assess the effect of the transfection-positive cell population, two separate lines of reasoning were utilized. First, to estimate the fraction of cells that contribute to transfection, we determined the proportion of cells that were successfully transfected. Second, to observe the cells that fail to contribute to protein expression, we determined the number of live/dead cells post-transfection. 

The transfection-positive cell population was estimated via EGFP reporter and by determining fluorescent-positive cells post-transfection. This assessment was first performed in adherent cells, and it was observed that the predictability for protein yields had further increased: now two top protein producers in CHO—NF51 and Reagent007—were among the top transfectors (Figure 1b vs. Figure 2c). However, some of the nonperforming protein producers were also flagged among the efficient transfection methods (such as jetOptimus in CHO in Figure 2c). Further analysis by dividing transfected cells into subpopulations of low, medium, and high EGFP+ did not offer additional insight (Figure S6).

Next, we investigated whether the transfection-positive cell count improves protein production prediction with the use of suspension culture —similar to what was observed in the case of total luciferase quantitation. The regular time period for protein production is approximately a week, therefore we wanted to confirm that the chosen pGFP reporter (which did not include any plasmid retention and replication enhancers) was expressed over several days. In both of the suspension cell lines the EGFP signals were present beyond five days (Figure S7a,b). Additionally, it was concluded that a 48 h end-point was the optimal timeframe for the assay in suspension cells (Figure S7c), as there was no significant increase in transfection-positive cell population after the 48 h mark. From transfection-positive cell count profiles (Figure 2d) it was observed that the highest transfection-positive percentages were achieved with CPP-based methods and PEI-Max in CHO cells, whereas in HEK cells the CPP NF71, Xfect, and jetOptimus were the top performers (Figure 2d).

**Figure 3:**
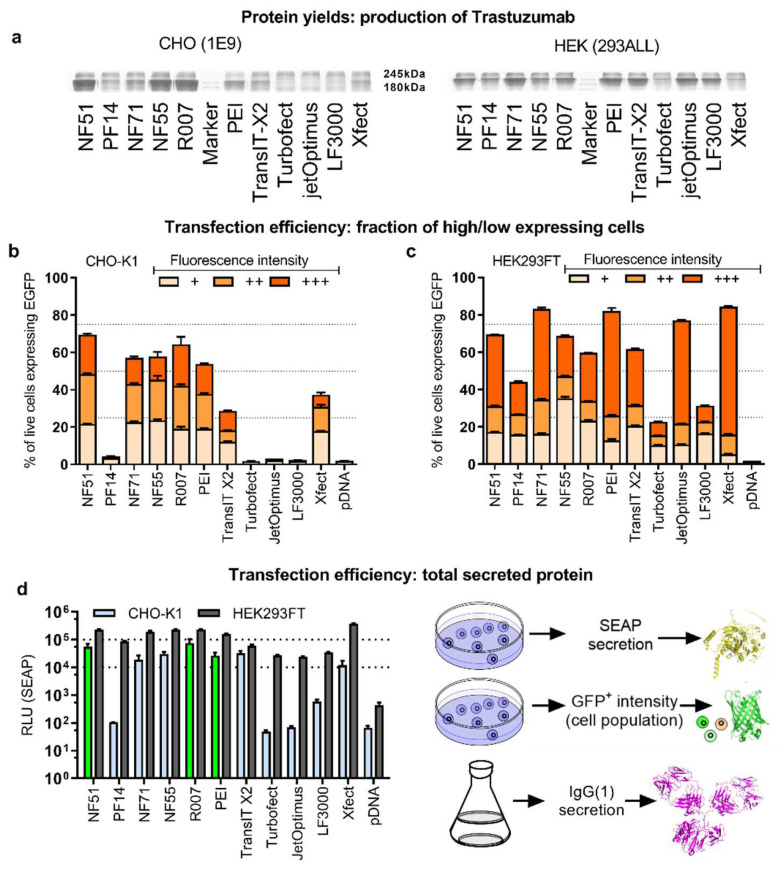
Using a suspension cell culture is a prerequisite for the prediction of protein production. (**a**) The trastuzumab mAb production in a suspension cell culture. 2 µg of pDNA was used per 6-well plate well. For CHO 1E9 cells assessment by SDS-PAAG 10%, Coomassie staining was performed 8 days post-transfection. For HEK293ALL analysis was performed 5 days post-transfection. (**b**–**d**) CHO-K1 or HEK293FT were cells transfected in serum-free media with 0.75 µg of pDNA. CPP/pDNA complexes were formed at CR2:1. For PEI N/P20 (HEK293FT) or N/P60 (CHO-K1) was used. 4 h post-transfection fresh media was added to the wells. Analysis was performed 48 h post-transfection. (**b**) CHO-K1 and (**c**) HEK293FT cells were transfected with pGFP. The fluorescent positive cell population was detected from the live cell population. The populations of fluorescent positive cells were divided into fractions of cells with low signal (+), medium signal (++), and strong signal (+++). (**d**) Cells transfected with pSEAP. RLU from SEAP-linked chemiluminescent assay were detected from collected media. N ≥ 3 for results shown in (**b**–**d**).

To analyze the impact of the efficacy assays (Figure 2) on the prediction of protein production, a second round of mAb production was performed to compare all the transfection methods. Moreover, it was done in CHO as well as HEK293 producer cells over a 1-week production cycle (Figure 3a). The superiority of the CPP transfection over polymeric and liposomal methods was observed in CHO cells (Figure 3a). The noted CPPs NF51, NF55 and R007 achieved a considerably higher level of the expressed protein, compared to all the other methods. The CPPs NF51 and NF55 were developed previously, and although their high efficacy for pDNA transfection has been shown [13,14], their utility in protein production settings as well as their superiority over polyplex and lipoplex approaches had not been explored before. 

In order to estimate the protein production predictive power, transfection-positive cell counts were compared to protein production efficacies (Figure 2d vs. Figure 3a, statistical analysis for all groups throughout the work are shown in Supplementary Table S2, and plots in Supplementary Table S3). It was observed that transfection-positive cell counts (Figure 2d) successfully flagged both the performers (highlighted in green) and non-performers in mAb production: NF51, NF55, Reagent007 and PEI-Max were among the best transfectors as well as the highest protein-producing groups (although PEI-Max cannot be considered on par with the CPP methods). Conversely, the rest of the methods lacked in transfection and mAb production. 

In HEK293, the protein was produced most effectively when transfected with the CPP NF71, followed by polyplex methods PEI-Max, TransIT, and jetOptimus (Figure 3a). The CPP NF71 was developed recently [16] and has been shown as an efficient siRNA and miRNA transfection reagent [16,19], but it has not been demonstrated in any of the pDNA-based applications before. However, in HEK cells neither the reporter assays (total protein or transfection-positive cell population) nor the culturing condition (adherent or suspension) were able to predict performance in mAb production, thus further investigation is needed for this cell line.

### 2.3. Expressed Protein Yields Are Predicted by the Total Secreted Protein Reporter

In order to establish whether specific subpopulations of cells with high- or low-expressed protein levels determine the outcome of the ultimate protein yields, the GFP+ cell populations were further investigated. The transfection-positive suspension cell subpopulations were divided into three subcategories: cells with low, medium, and high expressed protein levels (Figure 3b–c, Figures S8 and S9). However, none of these sub-fractions improved prediction precision, as the subpopulation proportions correlated primarily with the total GFP+ cells.

Considering that the ultimate goal lies in the effective production of large mammalian proteins that depend on processing in ER and Golgi, and are often secreted, a reporter protein that is also critically dependent on the ER and Golgi processing, and is secreted out of the cell—the secreted alkaline phosphatase (SEAP)—was included. The profiles of pSEAP transfection (Figure 3d) indicated that the SEAP expression in the CHO suspension culture correlated strongly with the mAb protein yields (r = 0.88, *p* < 0.05). In the HEK293 suspension culture, SEAP expression correlated with the number of GFP+ cells, but, unlike in CHO, neither of these efficacy assays correlated with mAb production.

### 2.4. High Viability of Cells Does Not Correlate with High Protein Expression

**Figure 4:**
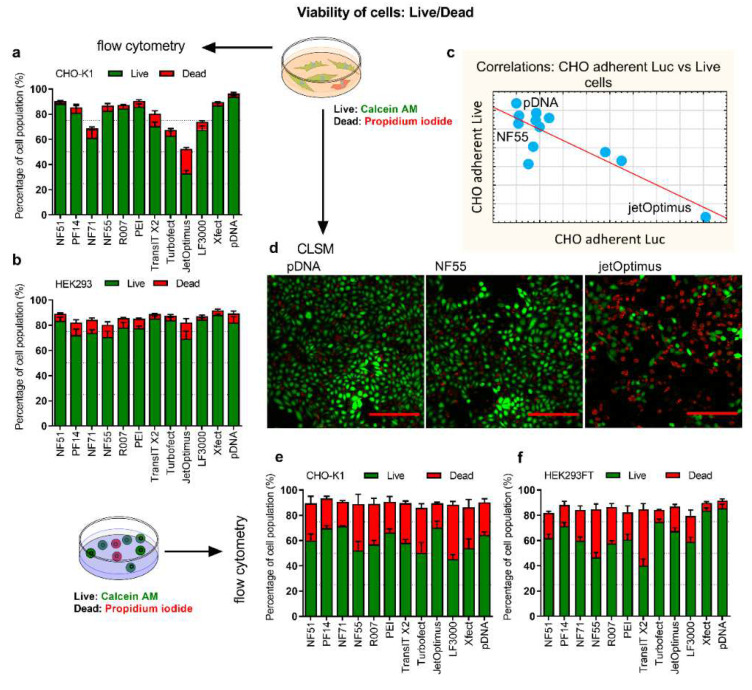
Cell viability post-transfection was assessed by the live/dead assay. Adherent (**a**) CHO-K1 cells and (**b**) HEK293 cells were transfected with 0.5 µg of pLuc per 24-well plate well. CPP/pDNA complexes were formed at CR3:1, and PEI complexes at N/P20. Analysis was done 24 h post-transfection. Calcein AM (live) and PI (dead) were added to detect live and dead cells. (**c**) The correlation graph depicting correlations between luciferase expression levels against viability of cells in adherent cell experiments. (**d**) The representative confocal images of cells 24 h post-treatment with pDNA, and cells transfected using jetOPTIMUS (jetOptimus) or NF55 (NF55). The red bar corresponds to 200 µm. Suspension (**e**) CHO K1 and (**f**) HEK293FT cells were transfected in serum-free media with 0.75 µg of pSEAP. CPP/pDNA complexes were formed at CR2:1, and PEI/pDNA at N/P20 (HEK293FT) or N/P60 (CHO-K1). 48 h post-transfection cells were collected, and Calcein AM and PI were added to detect live and dead cells from the whole cell population. N ≥ 3.

The successful expression of proteins is dependent on the number of viable cells. In order to characterize the cells that fail to contribute to transfection, the numbers of live and dead cells were analyzed. Although BrdU (Figure S10a) and MTS assays (Figure S10b) were considered, the live/dead assay was selected due to its high optimization range, compatibility with different detection methods, and because it reflected both live cells and cells with compromised cell membranes in a cell population (Figure 4a,b,d–f, Figures S11–S13). The aim was to analyze whether the proportion of (living) cells correlated with the expressed protein amount and if there was a higher number of live cells in the groups that performed well in protein production. 

We observed that in the CHO-adherent cells, some of the transfection methods were accompanied by an apparent reduction of viable cells. Controversially, the most “toxic” methods were concurrently very efficient transfection mediators in the adherent culture: the Pearson coefficient between adherent CHO total luciferase expression and the number of live cells was r = −0.85 (*p* < 0.05). Two different cases from this correlation were visualized with the CLSM: jetOptimus as the highest total luciferase transfection, and the least number of live cells. NF55 was one of the least performing in total luciferase expression in adherent CHO, but among the group with the highest number of viable cells (Figure 4c,d). In suspension cells, there was no strong correlation between cell viability (Figure 4e,f) and other assays.

On the one hand, we can conclude that reduced cell viability post-transfection may not mean much in adherent conditions, i.e., it is not necessarily a negative occurrence by itself. However, in the context of protein production in defined media, the total luciferase expression in adherent cell culture is not correlated with the protein yields. If anything, the correlation is negative (r = −0.59, but statistically not significant). 

### 2.5. Correlations between Protein Production and Screening Methods

We were most interested in finding connections that predict long-term and large-scale protein production efficacies from fast- and small-scale assays. To achieve this, we screened a number of methods that reflected different aspects of transfection efficacies and toxicities in two producer cell lines. We constructed a correlation matrix between the numerical outputs of all the assays for both the cell lines. The correlation matrix is presented in Supplementary Table S3.

**Figure 5:**
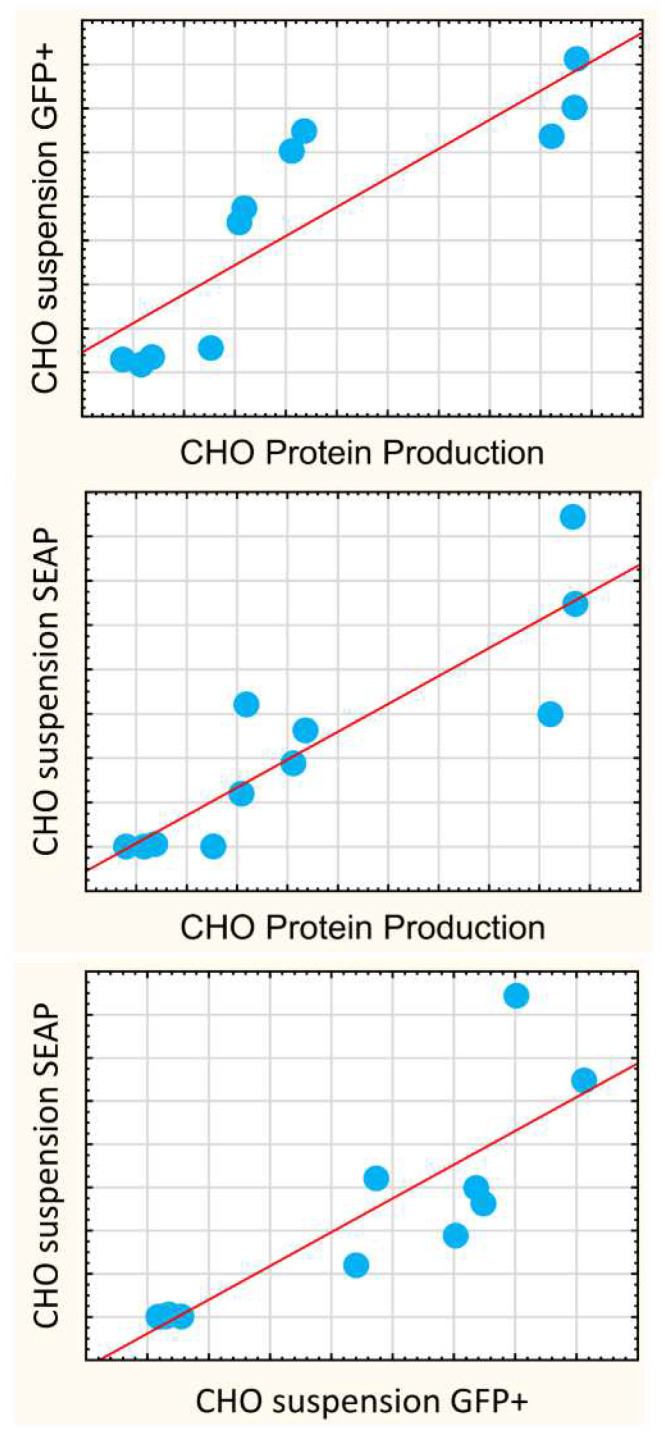
The protein production yield correlates with short-term assays of the secreted reporter protein and transfection positive population in CHO suspension culture. A scatterplot that highlights the correlations between the protein production yields and the assays of transfection-positive cell population (r = 0.86, *p* < 0.05) and SEAP protein secretion (r = 0.88, *p* < 0.05) in CHO suspension cells. The full correlation matrix with the Pearson coefficients is presented in Supplementary Table S3.

The most significant conclusions from the above are as follows. First, in CHO, the protein production yields correlated with both the suspension expressions of SEAP (r = 0.88, *p* < 0.05) and GFP+ population (Figure 5, r = 0.86, *p* < 0.05), but did not correlate with luciferase quantitation in neither adherent nor suspension media (Supplementary Table S3, Figure S15). This forced us to conclude a surprising implication: luciferase reporter quantitation assay, despite its excellent technical aspects, is not a good screening assay for predicting efficacies in protein expression. Conversely, the application of SEAP and GFP reporters should be encouraged as these create valuable data that can be used for protein production. 

The second implication is, as clearly illustrated in the correlation analysis (Supplementary Table S3), that the adherent culture methods generally fail to predict the effects of transfection in suspension conditions. Neither transfection efficacy assays, nor viability/toxicity assays correlated with protein production efficacies in CHO and HEK293. Hence, when working with the applications of protein expression in mammalian cells, suspension culturing is a must, even in research laboratory settings. 

Third, although the current report exemplifies several useful predictors for the CHO producer cell line in terms of efficacy assays and transfection methods, the utility and implications for the HEK293 are, unfortunately, less significant. Although we had demonstrated that a CPP-based transfection with NF71 significantly outperformed polyplex and lipoplex methods in protein production in HEK293, predictive transfection assays for this cell line should be explored in the future. 

Finally, in the current report, we presented cell-penetrating peptide-based transient transfection that significantly outperformed the most widely used method in protein production industrial settings, the PEI. We showed that NF55 and NF51 were efficient for CHO-based production, and NF71 is an excellent performer in HEK293 cells. These methods have the potential to replace PEI in industrial settings and offer higher yields for therapeutic protein production. Considering that the current report only intended to find correlative connections between various efficacy assays and transfection reagents, it offers little proof that would explain the mechanisms behind the observations. For example, it would be useful to mechanistically explain why the CPPs NF55, NF51 and R007 are significantly more efficient in protein expression than PEI or LF3000. With that knowledge, the process can be further improved. The mechanism for their success may be either increased efficacy in transfection, or less negative effects in cellular growth processes. More specifically, the process of association with the nucleic acid, interactions with the cell membrane, endosomal escape, long-term effects of cellular accumulation on cell viability are among the questions that should be answered in a follow-up study.

## 3. Materials and Methods

### 3.1. Transfection Reagents

Cell-penetrating peptides (CPP) NickFect51 (NF51), NickFect55 (NF55), NickFect71 (NF71), and PepFect14 (PF14) (Supplementary Table S1) were developed for in vitro and in vivo nucleic acid delivery, and represent four different design strategies for PF and NF CPP lines. NF51 was chosen as it efficiently delivers different nucleic acid cargoes into a variety of cell lines, including hard-to-transfect cells [14], both in serum-free and serum-containing conditions. The further development of NF51, with in vivo delivery in mind, CPP NF55, has an advantage in serum-containing conditions. It has been used for pDNA delivery with in vivo mouse models [13]. The choice of NF55 was based on its efficacy as a plasmid delivery vector. In order to gain the triggered release of delivered cargo, further carriers were developed—one of them was NF71 [16]. It has been shown to efficiently deliver nucleic acids into different cell lines, including hard-to-transfect cells [16,19]. PF14 is a CPP rich in ornithines and leucines, with a relatively high positive net charge. It has been used to efficiently deliver pDNA in vitro and in vivo [17].

CPPs were synthesized on an automated peptide synthesizer (Biotage Initiator+ Alstra, Biotage, Uppsala, Sweden) using the fluorenylmethyloxycarbonyl (Fmoc) solid phase peptide synthesis strategy with Rink-amide ChemMatrix resin (0.41 mmol g−1 loading) to obtain C-terminally amidated peptides. The fatty acid was coupled manually to the N-terminus of the peptide overnight, at room temperature with 5 eq. of fatty acid. For the synthesis of the NF51, NF71, and NF55, the Boc-L-Orn(Fmoc)-OH (Iris Biotech, Frankfurt, Germany) was used to continue the synthesis from the side-chain amino group. The reaction was carried out in DMF using HOBT/HBTU for manual or DIC/Oxyma for machine synthesis as coupling reagents, with DIEA as an activator base. The cleavage was performed with trifluoroacetic acid, 2.5% triisopropylsilane and 2.5% water for 2 h at room temperature. Peptides were purified by reversed-phase high-performance liquid chromatography on a C4 column (Phenomenex Jupiter C4, 5 μm, 300 Å, 250 × 10 mm) using a gradient of acetonitrile/water containing 0.1% TFA. The molecular weight of the peptides was analyzed by matrix-assisted laser desorption-ionization/time of flight mass spectrometry (Brucker Microflex LT/SH, Waltham, MA, USA). The concentration of the peptides was determined based on dilutions of accurately weighed substances and absorption of tyrosine, where applicable.

Commercially available transfection reagents included in this work are as follows. Linear polyethyleneimine-based polyethyleneimine HCl MAX (PEI MAX^®^), MW 40,000 from Polysciences (Warrington, PA, USA) Peptide-based transfection reagent Reagent007 from Icosagen (San Francisco, CA, USA). From Thermo Fisher Scientific (Waltham, MA, USA), two reagents, Lipofectamine™3000 (LF3000) Transfection Reagent as a lipid nanoparticle-based technology, and Turbofect as a lipid-based cationic polymer transfection reagent were chosen. Xfect™ from Takara Bio (Tokyo, Japan) was characterized as a biodegradable transfection polymer suitable for both CHO and HEK, and adherent or suspension cells. From Polyplus (Berkeley, CA, USA) jetOPTIMUS^®^ was chosen, which is characterized as a cationic transfection reagent suitable for transient and stable gene expression in mammalian cell culture. From Mirus Bio (Madison, WI, USA) TransIT X2^®^ was chosen, which is characterized as a non-liposomal polymeric transfection system.

### 3.2. Cell Culture Maintenance

Adherent CHO-K1 and HEK293 cells were grown in Dulbecco’s Modified Eagle’s Medium (DMEM). They were supplemented with 0.1 mM non-essential amino acids, 1.0 mM sodium pyruvate, 100 U/mL penicillin, and 100 mg/mL streptomycin. For complete media, 10% (final) fetal bovine serum (FBS) was added. For transfection, serum-free media was used. Cells were passaged regularly when the confluence of cells reached 80–90%. Cells were maintained in a humidified incubator at 37 °C, 5% CO_2_. Suspension CHO-K1 cells were gradually adapted to suspension culture. HEK293FT cells were grown as suspension cell cultures in Xell HEK TF media, which were supplemented with 100 u/mL penicillin, 100 mg/mL streptomycin and 6 mM GlutaMAX™. Cell viability was assessed daily and the cell density was reduced regularly. Cells were maintained in a humidified incubator at 37 °C, 8% CO_2_. Suspension CHO 1E9 and 293ALL cells were grown as a suspension culture. CHO 1E9 cells were grown in Xell CHO TF media. 293ALL cells were grown in BalanCD HEK293 or Xell HEK TF media. Media was supplemented with 4 mM GlutaMAX™. For cell counting, we used theCytoSMART cell counter accompanied by 0.4% trypan blue staining prior measurement. The multi-well plates with cell cultures used for experiments were incubated in a humidified incubator at 37 °C, 5% CO_2_ for adherent cell cultures and at 37 °C, 8% CO_2_ for suspension cell cultures. For Trastuzumab monoclonal antibody expression, cell cultures were incubated in a humidified incubator at 37 °C, 8% CO_2_ on an orbital shaker platform.

### 3.3. Transfection

Four different reporter plasmids were used throughout the work. Firefly luciferase encoding plasmid (pMC.BESPX-GLucFLuc2, referred as pLuc), pEGFP-C1 plasmid (pGFP) expressing green fluorescent protein, pCMV_SEAP (pSEAP) expressing secreted alkaline phosphatase, and Trastuzumab mAb expressing plasmid pLic2.1. SEAP expressing plasmid pSEAP was generated by substitution of eGFP coding sequence in pEGFP-C1 with sequence coding for SEAP in pLIVE^®^ In Vivo Expression/Reporter Vectors (Mirus MIR 5620). SEAP integration into pSEAP plasmid sequence was confirmed by sequencing. 

Plasmid doses and transfection volumes were defined under specific experiments. For CPP/pDNA complex formation, the diluted pDNA and peptide were mixed in water. Complexes were formed based on the theoretical charge ratio (CR) of positive charges from the peptide in excess of the negative charges from the pDNA backbone. The optimal CR throughout experiments was between two and three (W/V ratios in Appendix A.1). Commercial reagents were used according to the manufacturer´s recommendations for the transfection of adherent cells. For suspension cells, a defined pDNA dose was used for better comparison. For specifications of complex mixing refer to Appendix A.1. 

It should be noted that all herein-presented short-term transfection measurements (i.e., luciferase and GFP reporters) were conducted without any means of retaining the transfection positivity in daughter cells. Whereas, all the protein production experiments were conducted by using the QMCF technology that includes cell lines with EBNA/PyLT elements and transfection with compatible plasmid expression vectors.

### 3.4. Luciferase Reporter Protein Expression Detection from Cell Lysate

For reporter luminescence (expressed from pLuc) assessment in adherent cell culture, 10,000 cells per 96-well plate well were seeded in 100 µL of media one day prior to transfection. Shortly before transfection, cell media was replaced with 100 µL of serum-free DMEM media. Cells were transfected with 0.2 µg of pLuc per well (refer to Section 3.3). 4 h post-transfection the media was replaced with 100 µL of serum-containing media. 24 h post-transfection, the media was aspirated, cells were washed with 1 × PBS, and 30 µL of lysis buffer (0.1% Triton X100 in 1 × PBS) was added. For experiments with suspension cells, 30,000–35,000 cells per 96-well plate well in 100 µL of serum-free media were seeded 1 h prior to transfection. Cells were transfected with 0.1 µg of pLuc per well. 4 h post-transfection, 100 µL of fresh serum-free media was added to each well. 48 h post-transfection, ½ of the volume was removed from the top of the final volume of 200 µL after cell sedimentation, and to the rest, 50 µL of lysis buffer was added (0.1% Triton X100 in 1 × PBS). For both adherent cells and suspension cells, the cells were incubated for 20 min. in lysis buffer at 4 °C for cell disruption. From the lysate, 20 µL was transferred to a black frame white well 96-well plate for luminescence measurement after the addition of substrate in buffer (Appendix A.2). The luminescence signal was detected with GloMax^®^ 96 microplate luminometer equipped with GloMax^®^ 1.9.2 software (Promega, Madison, WI, USA). RLU values were converted to RLU/mg by normalization to total protein in cell lysate. For protein detection, a Pierce™ BCA Protein Assay Kit was used. 

### 3.5. Secreted Alkaline Phosphatase (SEAP) Expression Detection from Cell Media

For SEAP (expressed from pSEAP) detection, 600,000–750,000 suspension CHO-K1 or HEK293FT cells per 24-well plate well were seeded 1 h prior to the experiment in 500 µL of media. Cells were transfected in serum-free media with 0.75 µg of pSEAP; 4 h post-transfection 1:1 volume of fresh media was added to each well. 48 h post-transfection, 200 µL of cell suspension from each sample was collected into a microcentrifuge tube. Cell suspension was centrifuged (500× *g*, 5 min) to pellet the cells. Supernatant was collected and heated for 30 min at 65 °C and then cooled to room temperature. 25 µL of the sample was transferred to black frame white well 96-well plates for luminescence measurement. To each sample, 50 µL of HEPES buffer (10 mM, pH 7.4) and 25 µL of diluted CDP-Star™ substrate (1 mM to a final concentration 0.25 mM) was added. Luminescence was measured over a 25 min period until the signals equilibrated. The luminescence signal was detected with a GloMax^®^ 96 microplate luminometer equipped with GloMax^®^ 1.9.2 software (Promega). Results are shown as relative luminescence units (RLU).

### 3.6. Green Fluorescent Protein (GFP)-Expressing Cell Population Detection with Flow Cytometry

For flow cytometry experiments detecting transfected cell population (expression from pGFP) from adherent cells, 50,000 CHO-K1 or HEK293 cells per well were seeded in 500 µL of serum-containing media on a 24-well plate one day prior to transfection. Shortly before transfection, the media was replaced with 500 µL of fresh serum-free media. Cells were transfected with 0.5 µg of pDNA per well. 4 h post-transfection the media was replaced with 500 µL of serum-containing media. 24 h post-transfection, the media was removed, cells were washed with 1 × PBS and 0.25% trypsin-EDTA was added to detach the cells from the plate. After detachment, 1 × PBS supplemented with 1% FBS was added, and cells were analyzed by flow cytometry to detect cell population and fluorescence positive cells. 

For suspension CHO-K1 and HEK293FT cells, 600,000–750,000 cells per 24-well plate well were seeded in 500 µL of serum-free media 1 h prior to the experiment, and transfected with 0.75 µg of pGFP per well. 4 h post-transfection 1:1 volume of fresh serum-free media was added to each well. A 200 µL sample of cell suspension was collected 48 h post-transfection for analysis. 

For flow cytometry analysis, an Attune™ NxT Flow Cytometer equipped with Attune™ NxT Software 3.2.1 was used. For gating, the forward-scatter (FSC) and side-scatter (SSC) of untreated cells were used. Events with high (clumped cells) or low (debris, complexes, and media components) size were excluded. Untreated cells were used to set the GFP+ threshold (~1.5% of untreated were gated as GFP+). For detection, a 488 laser with 515–545 nm filter was used.

### 3.7. Live/Dead Cell Viability Assay

#### 3.7.1. Flow Cytometry

For Calcein AM and Propidium iodide (PI)-based viability assay in adherent cells, 50,000 CHO-K1 or 75,000 HEK293 cells were seeded per 24-well plate well in serum-containing media 24 h prior to transfection. Cells were transfected with 0.5 µg pLuc per well in 500 µL of serum-free media; 4 h post-transfection the media was replaced with 500 µL of serum-containing media. 24 h post-transfection the media was removed, and cells were washed with 1 × PBS. 0.05% trypsin-EDTA was added to detach cells from the plate. Calcein AM (0.4 µM on cells) and PI (1 µL per sample) diluted in 1 × PBS were added to the cell suspension 15 min prior to analysis. 

For suspension CHO-K1 and HEK293FT cells, 600,000–750,000 cells per 24-well plate well were seeded in 500 µL of serum-free media 1 h prior to experiment, and transfected with 0.75 µg of pSEAP per well. 4 h post-transfection 1:1 volume of fresh serum-free media was added. For suspension cells, 200 µL samples were collected from cells 48 h post-transfection. Samples were centrifuged (500× *g*, 5 min) and the supernatant discarded. The cell pellet was suspended in 250 µL of detection mix consisting of Calcein AM (2 µL of 4 mM stock) and propidium iodide (20 µL) in 14 mL of 1 × PBS. After 15 a min. incubation at room temperature, the cells were analyzed.

For both adherent and suspension cell experiments the forward-scatter (FSC) and side-scatter (SSC) of untreated cells were used for gating cell population. Events with high (clumped cells) or low (debris, complexes, and media components) size were excluded. The gating for live/dead cell population signals from untreated cells, cells incubated with either Calcein AM or PI, and cells pretreated with Triton X100 were used. For Calcein AM, a 488 laser with 515–545 nm filter was used. For PI, a 561 nm laser with 577–593 nm filter was used.

#### 3.7.2. Confocal Microscopy

For confocal microscopy, 25,000 adherent CHO-K1 cells were seeded on an 8-well Nunc™ Lab-Tek™ (Thermo Scientific™, Waltham, MA, USA) and incubated overnight. The media was replaced with serum-free media and cells were transfected with 0.25 µg of pLuc plasmid. 24 h post-transfection, the cells were washed and the media was replaced with phenol red-free DMEM. Confocal images were captured from live cells with Zeiss LSM710 (Carl Zeiss AG, Jena, Germany). For detecting live cells (Calcein AM), a 488 nm laser with 593–590 filter was used. For detecting dead cells (PI), a 561 nm laser with 593–712 filter was used. The images were taken with 20× magnification. For analysis, ZEN 3.0 software (Carl Zeiss Microscopy GmbH, Jena, Germany) was used.

### 3.8. Trastuzumab mAb Expression

For Trastuzumab monoclonal antibody expression, 3,000,000 CHO 1E9 cells in Xell CHO TF or 293ALL cells in Balan CD HEK293 media were seeded on a 6-well plate and 2 mL of media shortly prior to transfection. For transfection, 2 µg of Trastuzumab monoclonal antibody expressing pDNA was used per well. For CHO cells 6% of Xell Basic Feed was added one day post-transfection. On the third day post-transfection 10% of Xell Basic feed was added and cells were transferred to a 30 °C incubator. On the sixth day post-transfection, 10% of Xell Basic feed was added. On the 8th day post-transfection the experiment was terminated. For HEK cells 20% of Xell HEK TF media was added one day post-transfection. On the fourth day post-transfection 6% of Xell HEK TF was added. On the fifth day post-transfection the experiment was terminated.

### 3.9. Statistical Analysis

Statistical analyses of cell culture experiments were done on GraphPad Prism version 9.3.1 for Windows (Graphpad Software, CA, USA). All results are shown as a mean with the SEM of at least three separate experiments, if not indicated otherwise. Pearson correlations and one-way ANOVA were conducted with the Statistica application (Dell, Round Rock, TX, USA).

## 4. Conclusions

Transient transfection is a necessary tool that permits fast protein expression before determining the main lead for the stable expression and continuation with clinical development of therapeutic proteins. In this research, we aimed to improve the transfection process by introducing two improvements. 

First, we aimed to improve the translatability of microliter scale transfection into large-scale transient protein expression. We described small-scale transfection reporter assays that can predict protein yields. In production settings, it is difficult to pinpoint the exact bottleneck of low yields, as there are multiple possible variables that affect the efficacy: cell culture media and culturing conditions, transfection, and the properties of the protein-of-interest, among others. Having the correct transfection assessment tools allows the optimization of other parameters in the industrial production of proteins. 

Second, we characterized a new transfection method, based on cell-penetrating peptides, suitable for CHO and HEK293 cell lines that significantly outperform lipoplex approaches and the current industrial standard PEI. These peptides are new tools for industrial use and have the potential to replace PEI in transient protein expression. 

## Data Availability

All data are available upon reasonable request from the corresponding author.

## Supplementary Materials

The following supporting information can be downloaded at: https://www.mdpi.com/article/10.3390/pharmaceutics14091949/s1, Figure S1. Comparison of luminescence values when normalized to total protein form the cell lysates and without normalization. Figure S2. Optimization of complex formation conditions in adherent CHO-K1 and HEK293 cells assessed by total reporter luciferase quantitation from cell lysate; Figure S3. The pDNA dose and media change effect based on total reporter luciferase quantitation from cell lysate of transfected adherent CHO-K1 and HEK293 cells. Figure S4. Optimization of suspension cell culture transfection volume and time assessed by total reporter luciferase quantitation. Figure S5. Optimization of suspension CHO-K1 and HEK293FT cell transfection conditions assessed by total reporter luciferase quantitation. Figure S6. Fluorescence intensities of transfected adherent cell population expressing green fluorescent protein. Figure S7. Transfected suspension cell population expressing green fluorescent reporter protein. Figure S8. Flow cytometry profiles of transfected suspension CHO-K1 cells expressing green fluorescent protein. Figure S9. Flow cytometry profiles of transfected suspension HEK293FT cells expressing green fluorescent protein. Figure S10. Proliferation of cells post-transfection assessed by metabolic activity (MTS) and de novo synthesis assessment (brdU) in adherent CHO-K1 and HEK293 cells. Figure S11. Live/Dead assay control groups in adherent CHO-K1 and HEK293. Figure S12. Live/Dead assay on suspension CHO-K1 and HEK293FT cells analyzed 24 h and 48 h post-transfection. Figure S13. Live/Dead assay on suspension HEK293FT cells analyzed 24 h and 48 h post-transfection. Figure S14. Full SDS gel images used in Figure 3. Figure S15. Scatterplot that highlights the correlations between the protein production yields and the assays of transfection-positive cell population and SEAP protein secretion in HEK293 cells. Table S1. Cell-penetrating peptides used in this work, and their sequences. Tables S2 and S3. Statistical analysis of the transfection efficacy.

## Appendix A

### Appendix A.1. Methods. Transfection Complex Mixing Specifications per 1 µg of pDNA

For CPP/pDNA complexes, the mixing ratios per 1 µg of pDNA were defined as follows: For NF51, 1.5 µL (CR2) or 2.3 µL (CR3) of 1 mM peptide. For NF71, 1.3 µL (CR2) or 2 µL (CR3) of 1 mM peptide. For PF14, 1.2 µL (CR2) or 1.8 µL (CR3) of 1 mM peptide, and for NF55, 2 µL (CR2) or 3 µL (CR3) of 1 mM peptide. The complex was formed in 1/10th of the final transfection volume. The volume was adjusted by adding ultrapure water to pDNA prior to the addition of the peptide to the complex mix. Complexes were incubated 20–40 min. at room temperature before addition to the cells in media. For PEI-Max, the N/P ratio was used to calculate complex formation ratios. The N/P ratio reflects the ratio of polymer amine (N or nitrogen) groups to nucleic acid phosphate (P) groups. The reagent was diluted in water to a concentration of 1 mg/mL and was added to pDNA diluted in ultrapure water. The mix was vortexed for 5 s and incubated at room temperature for 20 min prior addition to the cells. For N/P20 2.6 µL, N/P30 3.9 µL and N/P60 7.8 µL of 1 mg/mL PEI was added per 1 µg of pDNA in ultrapure water. For Reagent007, the ratio per 1 µg of pDNA was defined as 4.75 µL of working solution mixed in ultrapure water. Complexes were incubated for 5 min at room temperature prior to addition to the cells. For Lipofectamine3000, the ratio per 1 µg of pDNA was defined as 2 µL of P3000 and 1.5 (low)–3 (high) µL of LF3000 reagent. For complex formation, pDNA and P3000 were mixed in a serum-free media (mix A). LF3000 was diluted in a serum-free media (mix B). Both components (A+B) were then mixed, suspended, and incubated at room temperature for 10 min prior to addition to the cells. For Turbofect, the ratio per 1 µg of pDNA was defined as 1–2 µL (2 µL used in this work) of reagent mixed in serum-free media. Plasmid was mixed with serum-free media, and then the reagent was added. Complexes were incubated at room temperature for 20 min. prior to addition to the cells. For Xfect, the ratio per 1 µg of pDNA was defined as 0.3 µL of reagent, mixed in the reaction buffer. Plasmid was mixed with a buffer, vortexed for 5 s, then the reagent was added, vortexed for 10 s and the complexes were incubated at room temperature for 10 min. prior to addition to the cells. For jetOPTIMUS, the ratio per 1 µg of pDNA was defined as 1 µL of reagent mixed in a reaction buffer. Plasmid was mixed with buffer, vortexed, reagent added, vortexed and incubated at room temperature for 10 min prior to addition to the cells. For TransIT X2, the ratio per 1 µg of pDNA was defined as 2-6 µL of reagent (3 µL was used in this work). Plasmid was mixed with the serum-free media and then the reagent was added. The complex solution was mixed by pipetting and incubated for 20 min. at room temperature prior to addition to the cells.

### Appendix A.2. Methods. Total Luciferase Quantitation and Whole Cell Lysate Potein Concentration Determination

Firefly luciferase was detected from the cell lysates of cells transfected with 0.1–0.2 µg of Firefly luciferase encoding pLuc. For adherent CHO-K1 and HEK293 cells, 10,000 cells per well were seeded in 100 µL of serum-containing media on a 96-well plate 24 h prior to transfection. Briefly prior to the transfection, the media was replaced with 100 µL of serum-free DMEM media. 4 h post-transfection the media was replaced with serum-containing media (if not stated otherwise), and further incubated. 24 h post-transfection the media was aspirated, cells were washed with 1 × PBS and lysis buffer was added to the wells. Lysis buffer consisted of 0.1% Triton X100 in 1 × PBS buffer. After 20 min. incubation, the lysate was mixed and used for further analysis.

For the suspension CHO-K1 and HEK293FT cells, 30,000–35,000 cells per well were seeded in 100 µL of serum-free media on a 96-well plate ~1 h prior transfection. For CHO-K1 cells Xell CHO TF media was used, and for HEK293FT cells Gibco Freestyle293 media was used. Both media were supplemented with GlutaMAX™. 4 h post-transfection 100 µL of fresh serum-free media was added to each well. 24–48 h post-transfection the cells were let to sediment and ½ of media was removed from the top. To the rest, 50 µL of lysis buffer was added and the cells were incubated for 20 min. for lysis. Lysate was gently mixed and used for further analysis.

Luminescence from expressed firefly luciferase was detected by mixing 20 µL of cell lysate with 100 µL of firefly luciferase detection solution. The detection solution was prepared shortly prior to addition to cells by mixing DTT (25 nM final concentration), D-Luciferin (1 mM), ATP (1 mM), Coenzyme A (25 µM), EDTA (1 mM), Tricine (20 mM), and MgCO3 (1 mM) in ultrapure water. After addition of the solution, the signal (relative luminescence units, RLU) was detected with GloMax^®^ 96 microplate luminometer equipped with GloMax^®^ 1.9.2 software (Turner BioSystems, Sunnyvale, CA, USA).

To determine total protein concentrations from the cell lysate, Pierce™ BCA Protein Assay Kit was used. Cell lysate was transferred to a transparent 96-well plate, and to each sample, 100 µL of solution consisting of component A and component B mixed in a 50:1 ratio was added. Following 30 min incubation, absorbance was measured at 562 nm. Absorption values were converted to protein concentrations using a standard curve generated by measuring samples with known protein concentrations. Absorption was measured with Tecan Sunrise microplate absorbance reader (Tecan Austria GmbH, Grödig, Austria). RLU values were converted to RLU/mg by normalization to the total protein in the cell lysate.

### Appendix A.3. Methods. MTS-Based Proliferation Assay in Adherent Cell Culture

10,000 CHO-K1 and HEK293 cells per well were seeded 24 h prior transfection on a 96-well plate in 100 µL of serum-containing phenol-free DMEM media supplemented with 1 mM sodium pyruvate, 0.1 mM non-essential amino acids, 100 U/mL penicillin, and 100 mg/mL streptomycin. Shortly prior to the transfection, the media on cells was replaced with 100 µL of serum-free media and complexes containing 0.2 µg of pLuc pDNA per well were added to cells. CellTiter 96^®^ AQueous One Solution Cell Proliferation Assay (MTS) was used as indicated by the manufacturer´s instructions. Briefly, 20 h post-transfection 20 µL CellTiter 96^®^ AQueous One Solution Reagent was added to each well and cells were further incubated for 4 h in a humidified, 5% CO_2_ atmosphere at 37 °C. Following incubation, absorbance was measured at 490 nm using a Tecan Sunrise microplate absorbance reader (Tecan Group Ltd., Mannedorf, Switzerland) to detect soluble formazan produced by cellular reduction of MTS.

### Appendix A.4. Methods. BrdU (De Novo DNA Synthesis) Proliferation Assay

10,000 CHO-K1 and HEK293 cells were seeded 24 h prior to transfection on a 96-well plate in 100 µL of serum-containing phenol-free DMEM media described above. Shortly prior to transfection, the media was replaced with 100 µL of serum-free media and complexes containing 0.2 µg of pLuc were added to each well. 4 h post-transfection the media was replaced with serum-containing media. Abcam BrdU Cell Proliferation ELISA Kit (colorimetric) was used as indicated in the manufacturer´s instructions. 24 h post-transfection the media was removed, and 200 µL of fixing solution was added to the cells. The plate was incubated at room temperature for 30 min. The fixing solution was aspirated and the cells were washed three times with 200 µL of wash buffer. To each well 100 µL of anti-brdU monoclonal antibody solution was added, and the cells were incubated at room temperature for 1 h. After removing the antibody solution, the cells were washed three times with 200 µL of wash buffer. To each well 100 µL of 1 × peroxidase goat anti-mouse IgG conjugate solution was added and incubated at room temperature for 30 min. The solution was then aspirated and the cells were washed three times with 200 µL of wash buffer. Subsequently, the plate was flooded with ultrapure water and tapped dry. To each well 100 µL of TMB peroxidase substrate was added and incubated in the dark, at room temperature for 30 min. Reaction was then stopped with 100 µL of stop solution. The absorbance from wells was measured at 450/550 nm with Tecan Sunrise microplate absorbance reader (Tecan Group Ltd., Bern, Switzerland).
