# Supplementary material for: Predicting Transiently Expressed Protein Yields: Comparison of Transfection Methods in CHO and HEK293
Source: Pharmaceutics. 2022 Sep 14;14(9):1949. doi: 10.3390/pharmaceutics14091949 (PMC9505259; doi:10.3390/pharmaceutics14091949)
Supplement: Supplementary file 1 [file pharmaceutics-14-01949-s001.zip › pharmaceutics-1848805-supplementary.pdf]

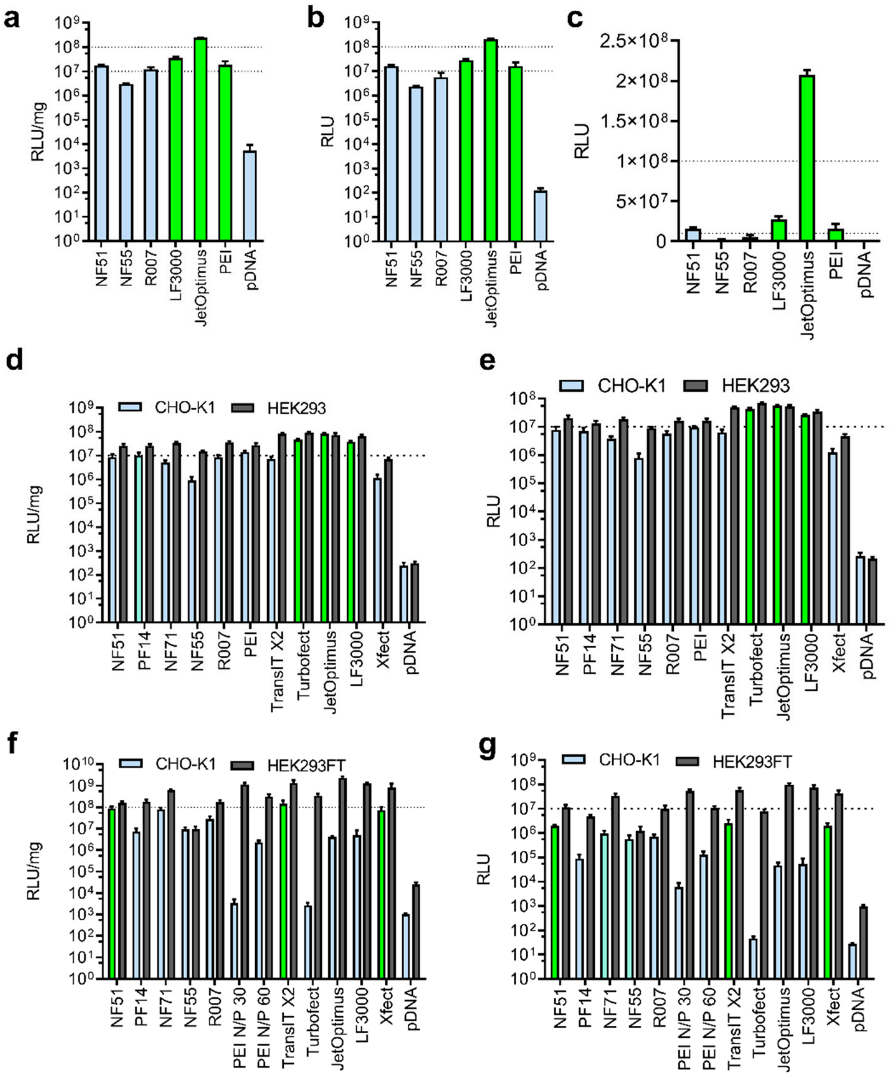

Figure S1. Comparison of luminescence values when normalized to total protein form the cell lysates and without normalization. Adherent CHO-K1 or HEK203 cells (a,b,c,d,f) or suspension CHO-K1 or HEK293FT cells (e,g) were transfected with 0.2 μg of Firefly luciferase encoding pLuc per well on a 96-well plate. Transfection was done in serum free media. Luminescence was measured from the cell lysate 24 h post-transfection. RLU was normalized to protein in figures a, d and f. Figure a to c are from the same experiment, but in figure a the result are shown as RLU/mg, in b as RLU and in c in RLU and the y-axis is in linear scale.

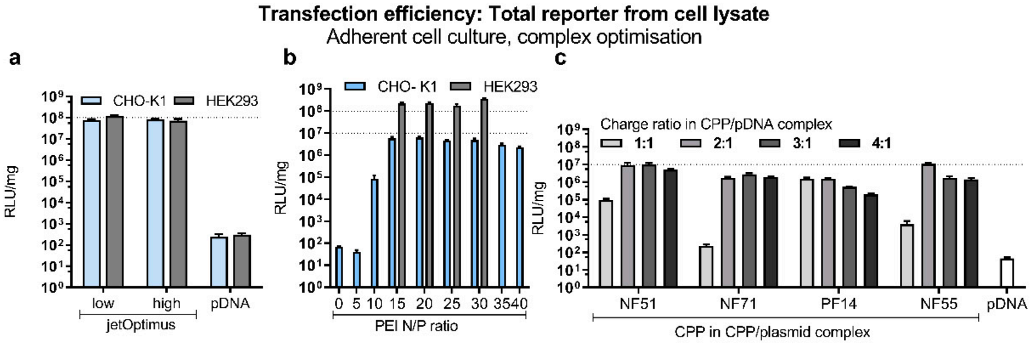

Figure S2. Optimization of complex formation conditions in adherent CHO-K1 and HEK293 cells assessed by total reporter luciferase quantitation from cell lysate. Adherent CHO-K1 or HEK293 cells were transfected with 0.2 μg of Firefly luciferase encoding pLuc per well on a 96-well plate. Transfection was done in serum free DMEM media. Luminescence was measured from the cell lysate 24 h post-transfection and relative luminescence units (RLU) were normalized to protein measured from the lysate. a) For jetOPTIMUS the amount of reagent was given as a range of reagent per 1 μg of pDNA. The lowest amount (low) and highest amount (high) of reagent per pDNA dose were tested. b) For PEI the optimization range was not indicated within the user protocol, therefore we tested a broader range based on theoretical PEI/pDNA N/P ratios. The N/P 20 was chosen for following experiments in adherent cells. c) CPP/pDNA charge ratio screen in adherent cells in serum free media. The charge ratio reflects the ratio between positive charges in the peptide and negative charges in the DNA phosphate backbone. Complexes formed with CPP in excess. On average, depending on the specific experiment, the optimal range was between CR2 and CR3.

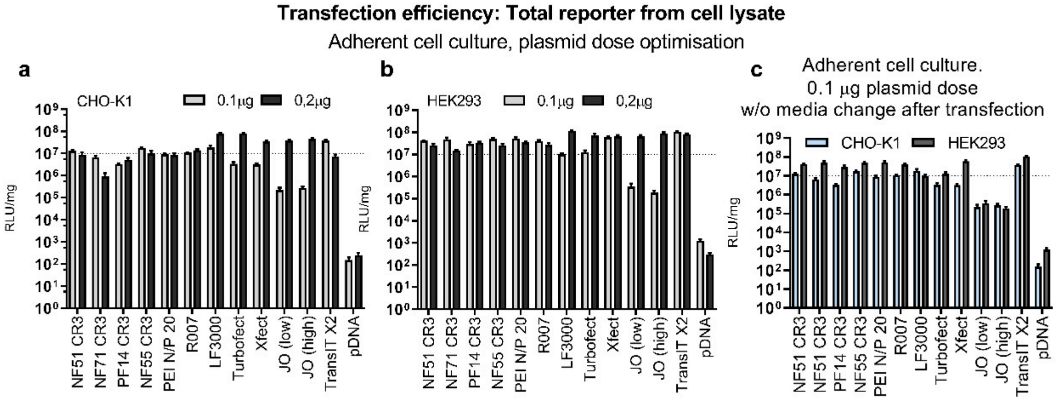

Figure S3. The pDNA dose and media change effect based on total reporter luciferase quantitation from cell lysate of transfected adherent CHO-K1 and HEK293 cells. Adherent CHO-K1 and HEK293 cells were transfected with Firefly luciferase encoding plasmid pLuc. Transfection was done in serum free media. Plasmid dose comparison in a) CHO K1 or b) HEK293 cells. Cells were transfected with either 0.1 μg or 0.2 μg pDNA dose per 96-well plate well in 100 μl of serum free media. 4 h post-transfection media was replaced with serum containing media. 24 h post-transfection cells were lysed and luminescence measured from the cell lysates. Same lysate was used to determine total protein concentration and RLU was normalized to protein in lysate. c) For CPP/pDNA complexes we often use transfection without media change 4 h post-transfection. We compared the transfection efficacy between transfection reagents and CPPs in this setting. The pDNA dose per well was 0.1 μg and transfection was done in serum free media. Media was not changed after addition of complexes, and cells were incubated with complexes in serum free media for 24 h. Following wash and cell lysis, the luminescence and protein concentration were determined from the lysate. Results are expressed as relative luminescence units per mg of total protein (RLU/mg). JO – jetOptimus

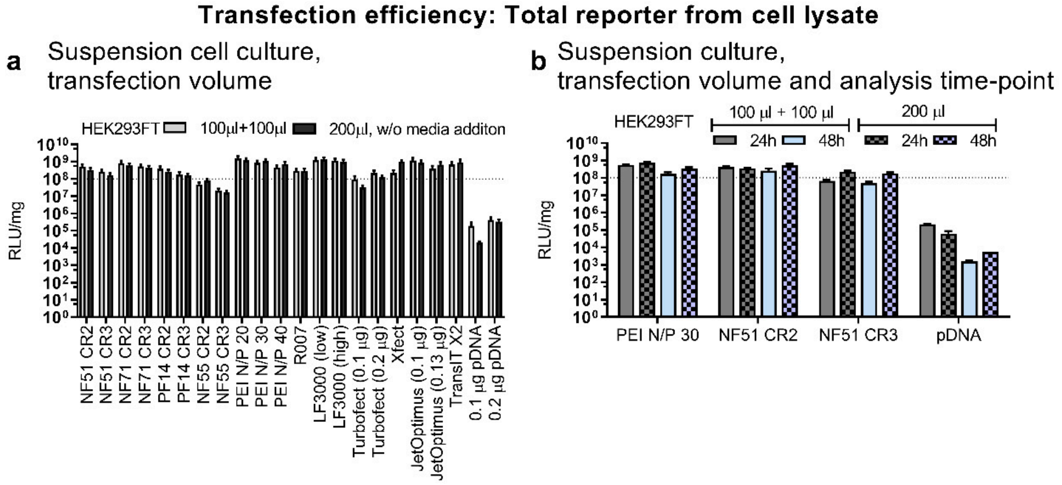

Figure S4. Optimization of suspension cell culture transfection volume and time assessed by total reporter luciferase quantitation. Suspension HEK293FT cells were transfected with 0.1 μg of Firefly luciferase encoding pLuc. To determine whether or not the addition of media would be beneficial to the transfection two setting were compared. With media addition 4 h post-transfection (100 μl + 100 μl), and transfection in the same final volume, but without addition of fresh media post-transfection (200 μl) . a) 24 h post-transfection cells were let to sediment and 100 μl of media was removed from the top. To each well 50 μl of lysis buffer (0.1% Triton X100 in 1 x PBS) was added to the remaining 100 μl of media with cells. After lysis 20 μl sample was used to determine RLU. Generally, there were no major differences between transfection efficacies between these two groups. b) Considering the time-point after transfection we screened some groups with the previous settings (100 μl + 100 μl vs 200 μl) at 24 h and 48 h post-transfection. Although the luminescence values for transfected groups did not significantly differ between 24h and 48h measurements, interestingly, the background signal in pDNA treated and untreated groups was lower 48h post-transfection. Compelled by more favorable signal-to-noise ratio we chose 48h post-transfection measurements for suspension cells. For CPP/pDNA complexes CR2 had a slightly higher reporter levels when compared to CR3, therefore we continued with CR2 in suspension cell cultures.

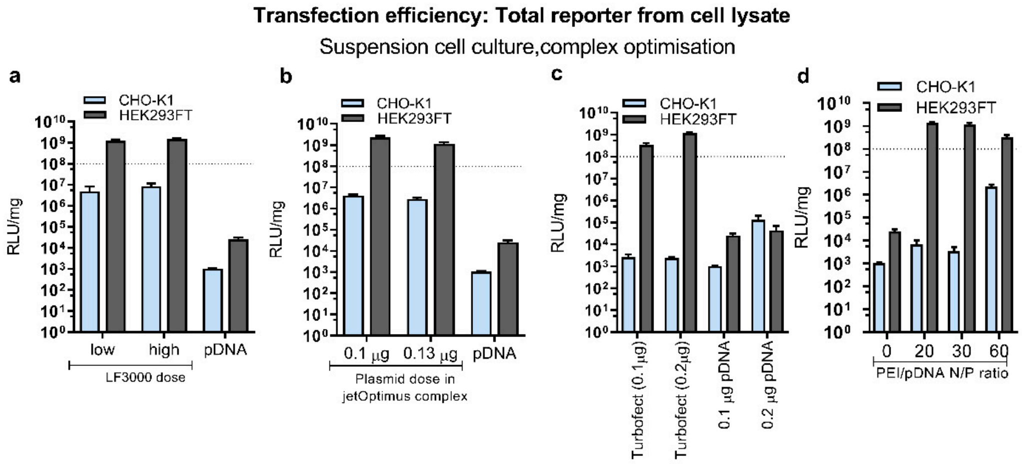

Figure S5. Optimization of suspension CHO-K1 and HEK293FT cell transfection conditions assessed by total reporter luciferase quantitation. Suspension CHO-K1 and HEK293FT cells, transfected with 0.1 µg (if not stated differently) Firefly luciferase encoding pLuc. 4 h post-transfection equal volume of media was added to each well. 48 h post-transfection 100 µl of media was removed, lysis buffer added and from lysate luminescence (RLU) and total protein concentration(mg) were determined. We tested a) LF3000 reagent dose, b) pDNA dose for jetOPTIMUS, c) PEI N/P ratio from 0 to 60, and d) pDNA dose for Turbofect in CHO-K1 and HEK293FT cells. Using higher reagent dose in LF3000/pDNA complexes did not have a significant effect, therefore for further experiments low dose was used in suspension cells. In case of jetOPTIMUS the indicated lowest pDNA dose was 0.13 ug, but also the 0.1 ug pDNA dose did not result in significantly lower signal. In PEI complexes, interestingly for CHO-K1 transfection, the required N/P ratio compared to HEK293FT cells was considerably higher, whereas in HEK293FT cells already the complexes formed at N/P 20 resulted in high reporter expression. In case of Turbofect we also included group with 0.2 µg pDNA dose, as this was indicated minimal amount of pDNA per 96 wp well.

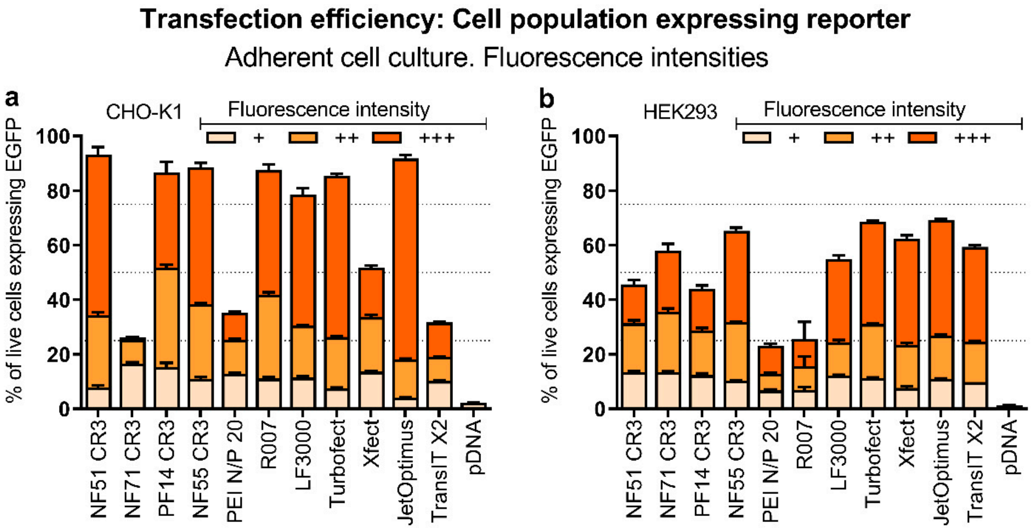

Figure S6. Fluorescence intensities of transfected adherent cell population expressing green fluorescent protein. 24 h prior transfection 50,000 cells per well were seeded on a 24-well plate well in 500 µl of media. Shortly prior transfection media was replaced with serum free media and adherent a) CHO-K1 and b) HEK293 cells were transfected with 0.5 µg of green fluorescent protein expressing pGFP. 4 h post-transfection media was replaced with 500 µl of serum containing media. 24 h post-transfection cells were washed, detached from the plate and cells with fluorescent signal were determined using flow cytometry. For gating untreated cells side-scatter and forward scatter plots were used, and from the cell population the threshold was set to ~1% fluorescent cells in untreated cell samples.

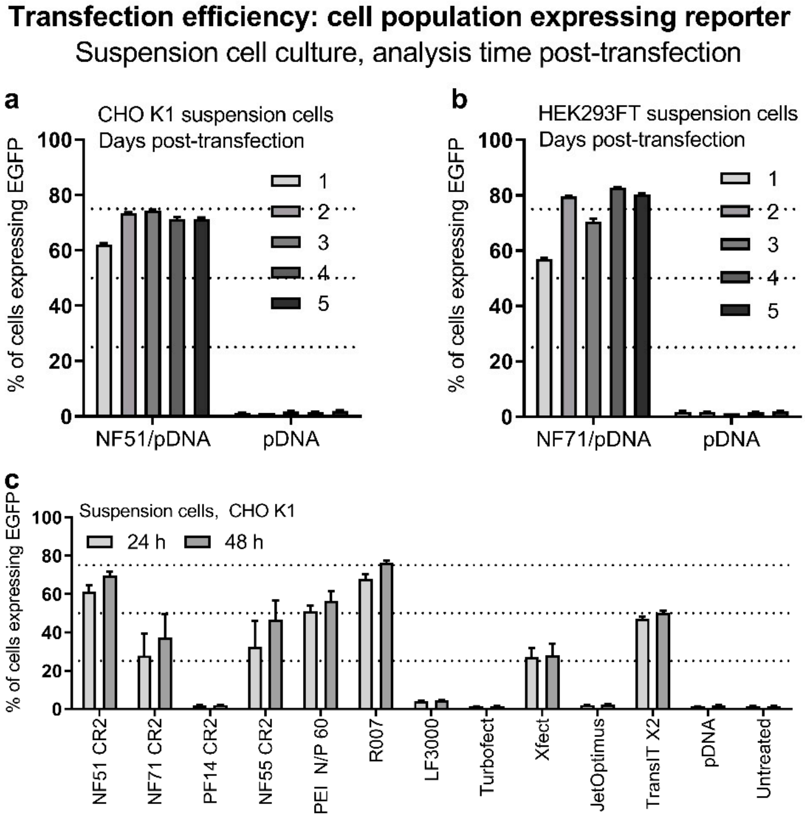

Figure S7. Transfected suspension cell population expressing green fluorescent reporter protein 1 h prior transfection 600,000-750,000 suspension cells per well were seeded on a 24-well plate in 500 µl of serum free media. For CHO cells Xell CHO TF media was used, and for HEK293FT cells Gibco Freestyle293 media was used. Both media were supplemented with GlutaMAX. The a) CHO-K1 and b) HEK293FT cells were transfected with CPP/pDNA complexes. 0.75 µg of green fluorescent protein encoding plasmid pGFP was used per well. As a control wells with cells treated with pDNA at the same dose were used. Reporter protein (GFP) expressing cell population was detected by flow cytometry 1 to 5 days post-transfection. c) The percentage of green fluorescent protein expressing cell population detected from suspension CHO-K1 cells 24 h and 48 h post-transfection of 0.75 µg of pGFP.

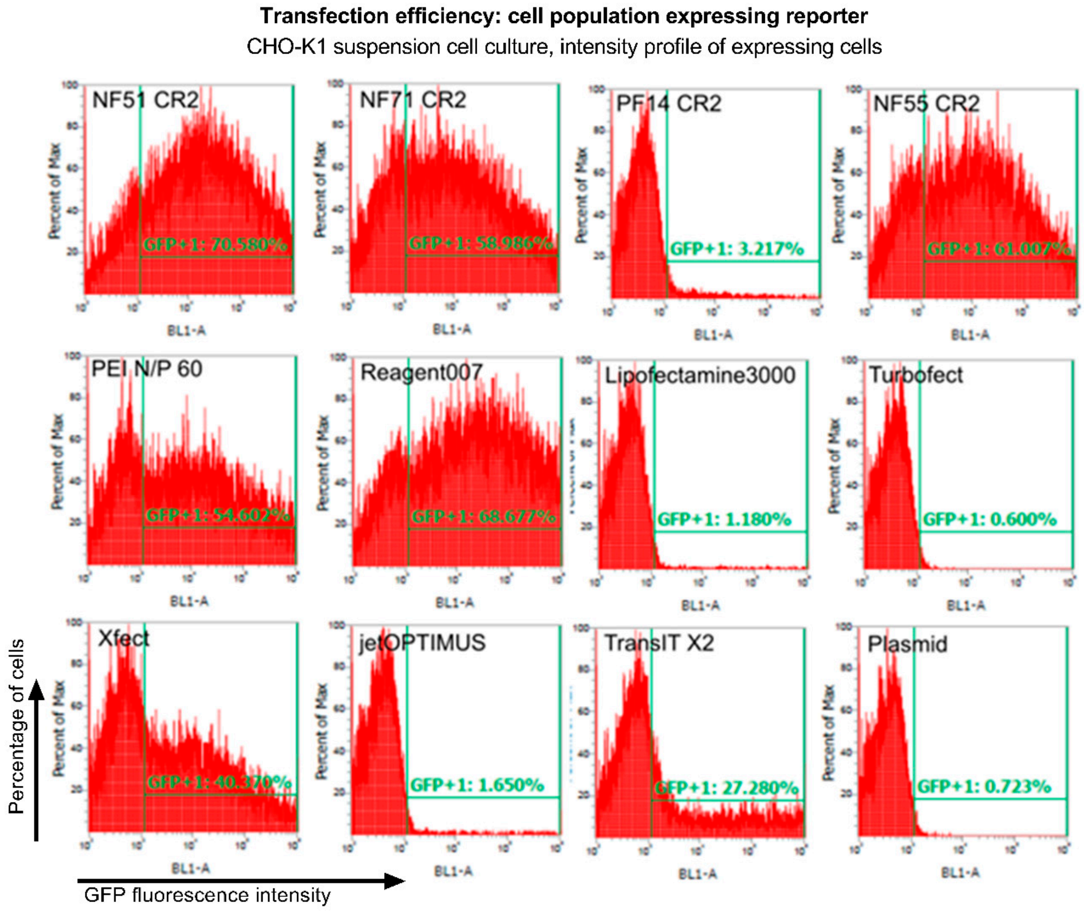

Figure S8. Flow cytometry profiles of transfected suspension CHO-K1 cells expressing green fluorescent protein. 600,000-750,000 suspension CHO-K1 cells per well were seeded on a 24-well plate in 500 µl of serum free Xell CHO TF media 1 h prior transfection. Cells were transfected with 0.75 µg of green fluorescent protein encoding pGFP per well. 4 h post-transfection 500 µl of fresh media was added to cells. 48 h post-transfection cells were analyzed by flow cytometry. The signal intensities from cells and percentages from cell population are shown on each graph. Marked on each is the transfection reagent used for transfection. GFP+ is showing gated events with signal intensity over threshold of ~1% of untreated cells.

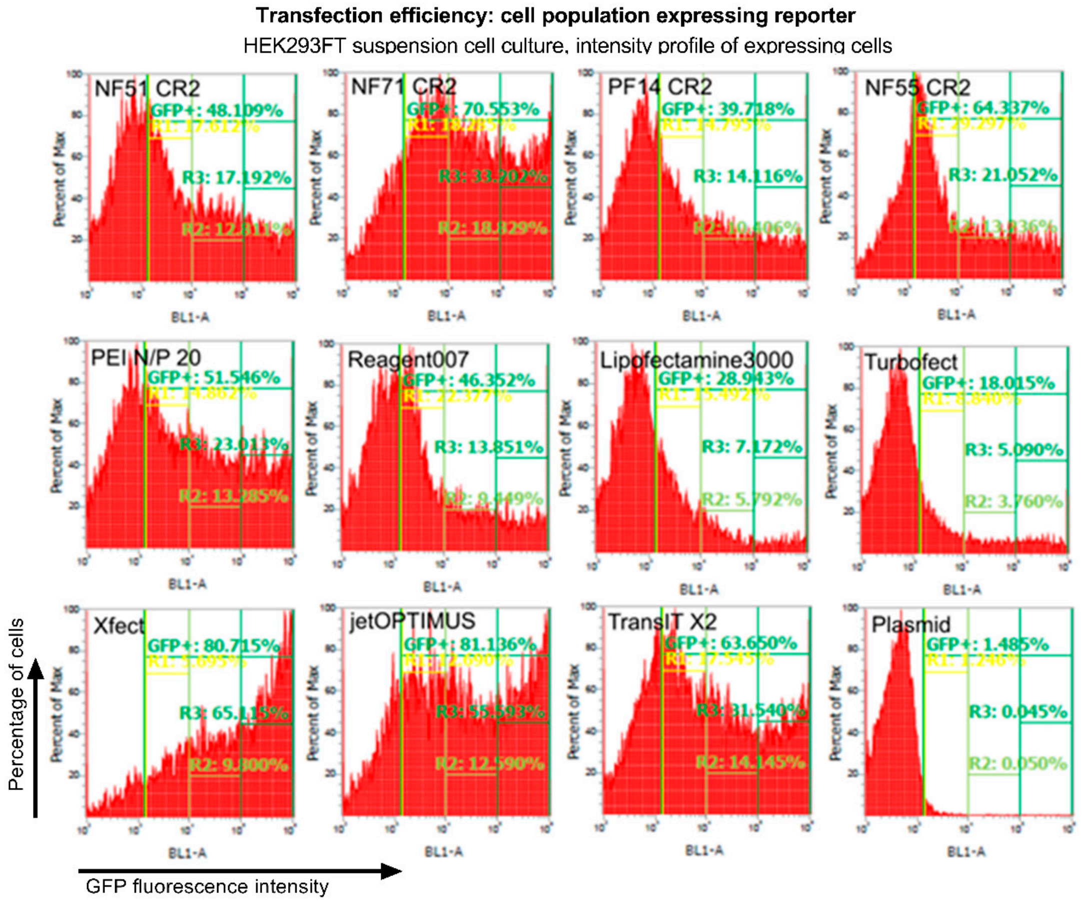

Figure S9. Flow cytometry profiles of transfected suspension HEK293FT cells expressing green fluorescent protein categorized to low, medium and high expressing cell populations. 600,000-750,000 suspension HEK293FT cells per well were seeded on a 24-well plate in 500 µl of serum free Gibco Freestyle293 media 1 h prior transfection. Cells were transfected with 0.75 µg of green fluorescent protein encoding pGFP per well. 4 h post-transfection 500 µl of fresh media was added to cells. 48 h post-transfection cells were analyzed by flow cytometry. The signal intensities from cells and percentages from cell population are shown on each graph. GFP+ is showing gated events with GFP signal.

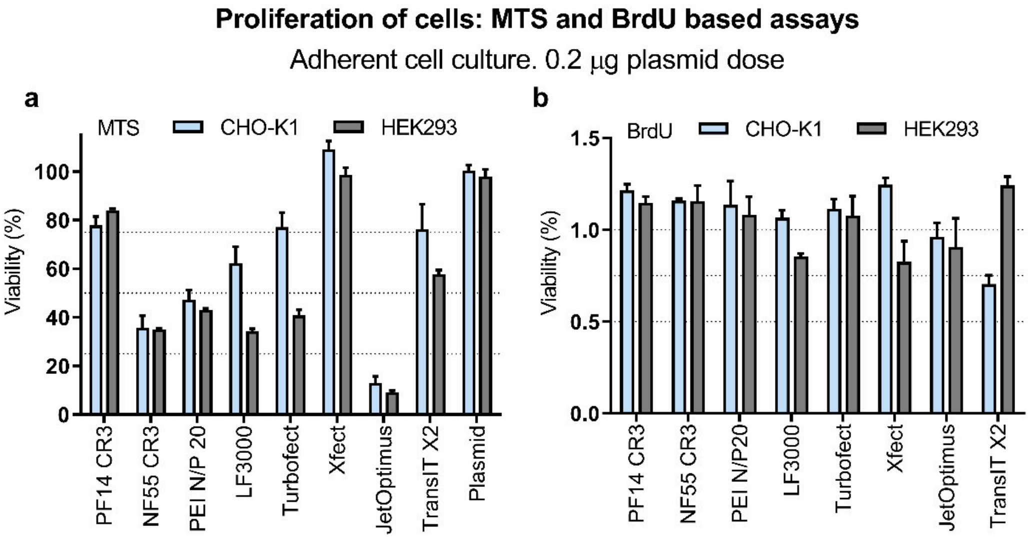

Figure S10. Proliferation of cells post-transfection assessed by metabolic activity (MTS) and de novo synthesis assessment (brdU) in adherent CHO-K1 and HEK293 cells. a) Cells transfected in 100 μl of serum free media with 0.2 μg of Firefly luciferase encoding pLuc per 96-well plate well. 20 h post-transfection 20 μl of CellTiter 96® AQueous One Solution Reagent was added to each well and cells further incubated at humidified incubator for 4 h, following detection of absorbance. Results are shown absorbance values normalized to untreated cells and converted to percentages, where absorbance from untreated cells (100%) and background (0%) was taken into account. (Details in Supplementary methods 3) b) BrdU assay, which reflects the proliferation of cells by incorporation into newly synthesized DNA. Abcam brdU Cell Proliferation ELISA colorimetric kit was used to determine cell proliferation 24 h post-transfection. Results are expressed as fold to untreated (1.0). (Details in Supplementary methods 4). The comparison between MTS and BrdU with the other assays did not yield useful and significant correlations and are not shown in the summary correlation matrix.

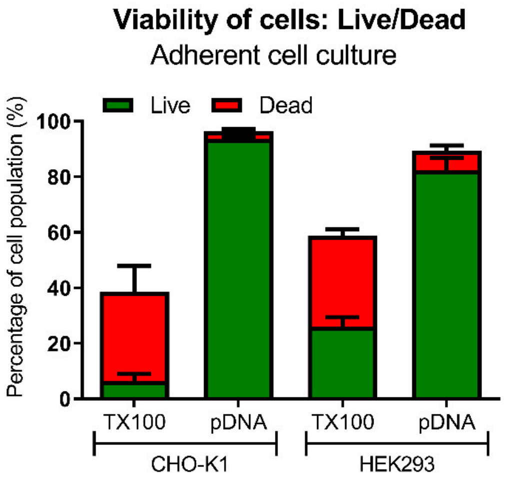

Figure S11. Live/Dead assay control groups in adherent CHO-K1 and HEK293. 50,000- a) CHO-K1 and 75,000 b) HEK293 cells seeded 24 h before transfection on a 24-well plate in serum containing DMEM media. Cells were incubated in serum free media with or without 0.5 µg of pDNA pLuc per well. 4 h post-addition of pDNA, the cell media was replaced with serum containing media. 24 h post-transfection cells were washed, detached from the plate and Calcein AM (Live) and PI (Dead) were added. For TX100 group with detection mix 0.1% Triton X100 solution was added to the cells. Flow cytometry was used to detect Live and Dead cells from cell population. Results are expressed as percentage of events from all events with FSC and SSC similar to cells.

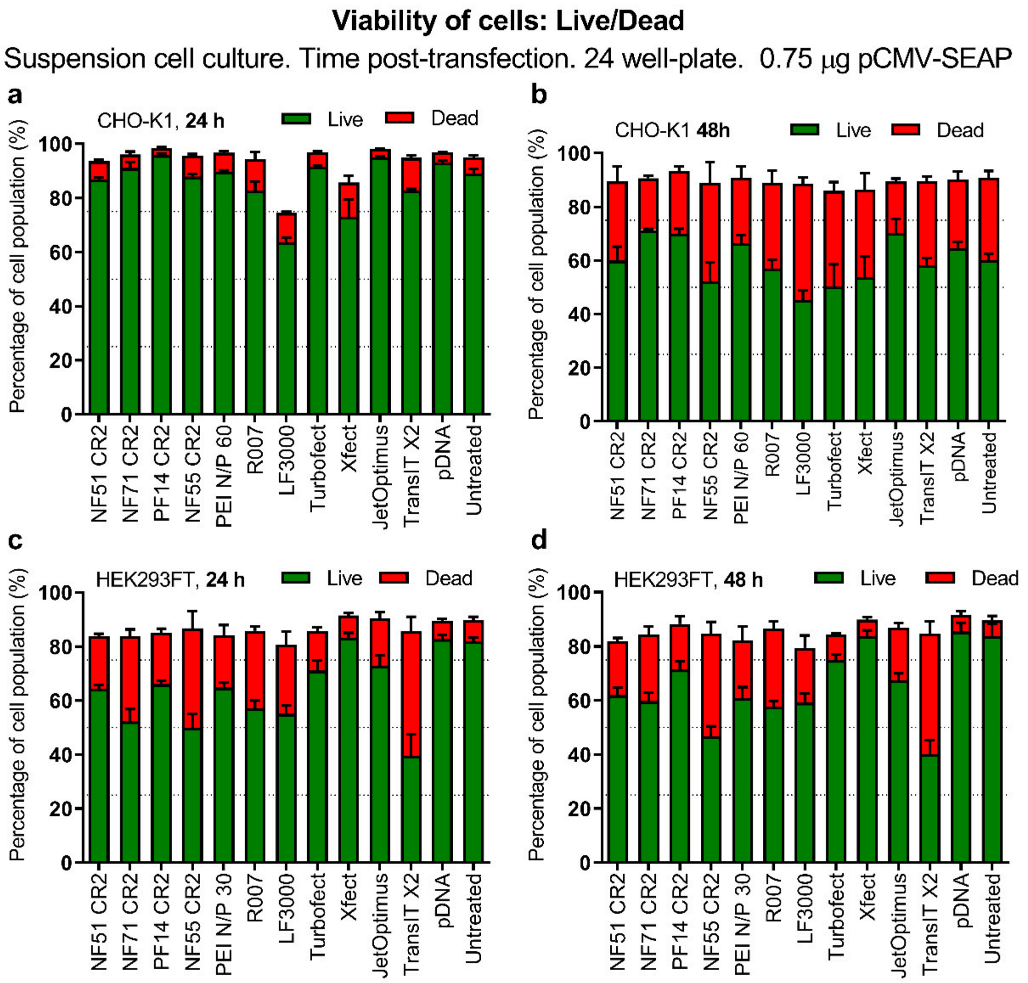

Figure S12. Live/Dead assay on suspension CHO-K1 and HEK293FT cells analyzed 24 h and 48 h post-transfection. 600,000-750,000 suspension a) and b) CHO K1 or c) and d) HEK293FT cells per 24-well plate well were seeded ~1 h prior transfection in 500 μl of serum free media. Cells were transfected with 0.75 μg of pSEAP per well. 4 h post-transfection 500 μl of fresh media was added. a) and c) 24 h, or b)and d) 48 h post-transfection cells were collected, centrifuged and solution containing Calcein AM and PI was added to detect Live (Calcein AM) and Dead (PI) cells from cell population with flow cytometry

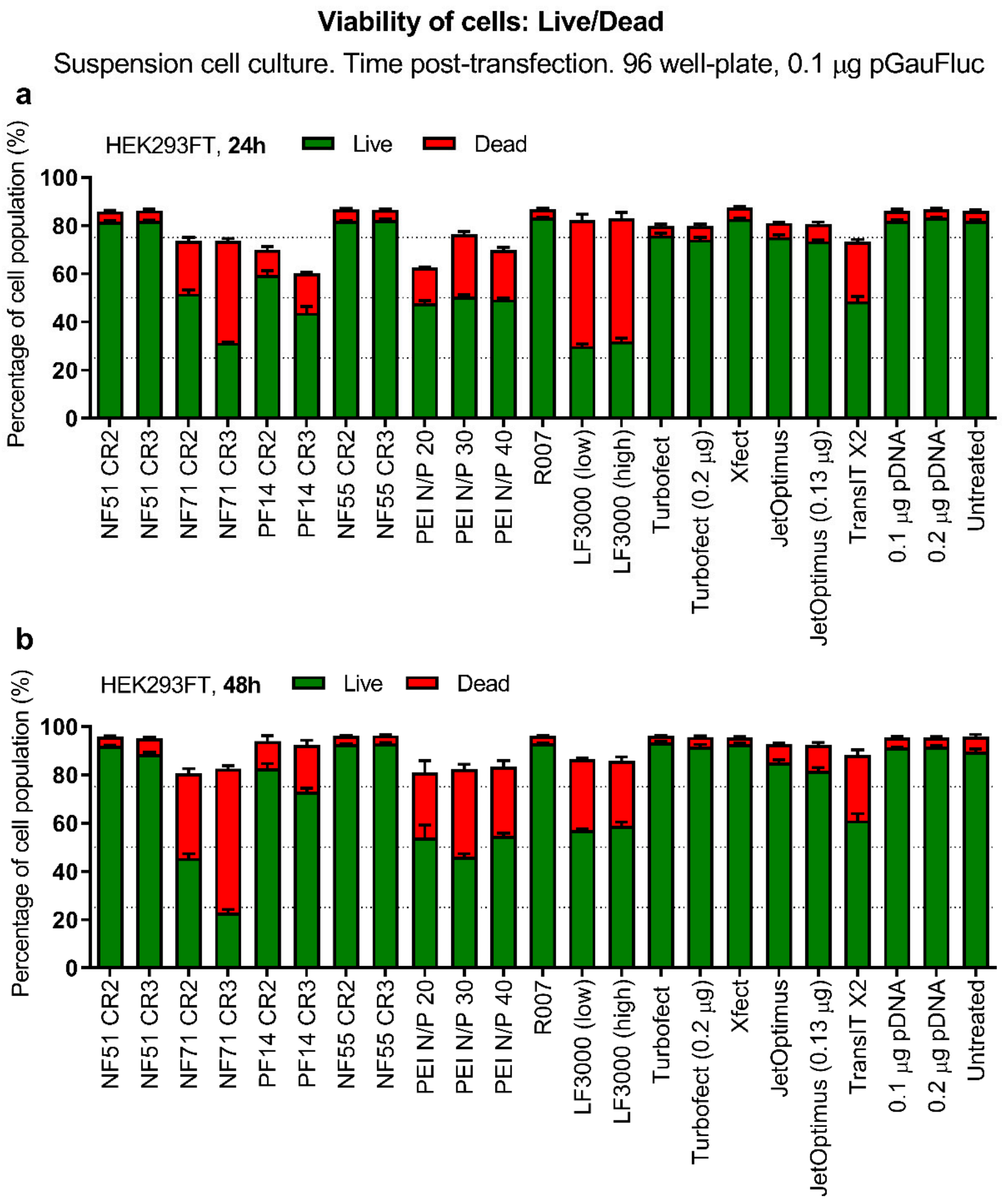

Figure S13. Live/Dead assay on suspension HEK293FT cells analyzed a) 24 h and b) 48 h post-transfection. For 96 well plate format 40,000 HEK293FT suspension cells per well were seeded in 100 µl of media per well 1 h prior transfection. Cells were transfected with 0.1 µg of Firelfly luciferase encoding pLuc. 4 h post-transfection 100 µl of fresh media was added. 24 h and 48 h post-transfection from 200 µl cell suspension 100 µl was collected and Calcein AM and PI was added to detect Live (Calcein AM) and Dead (PI) cells from cell population by flow cytometry.

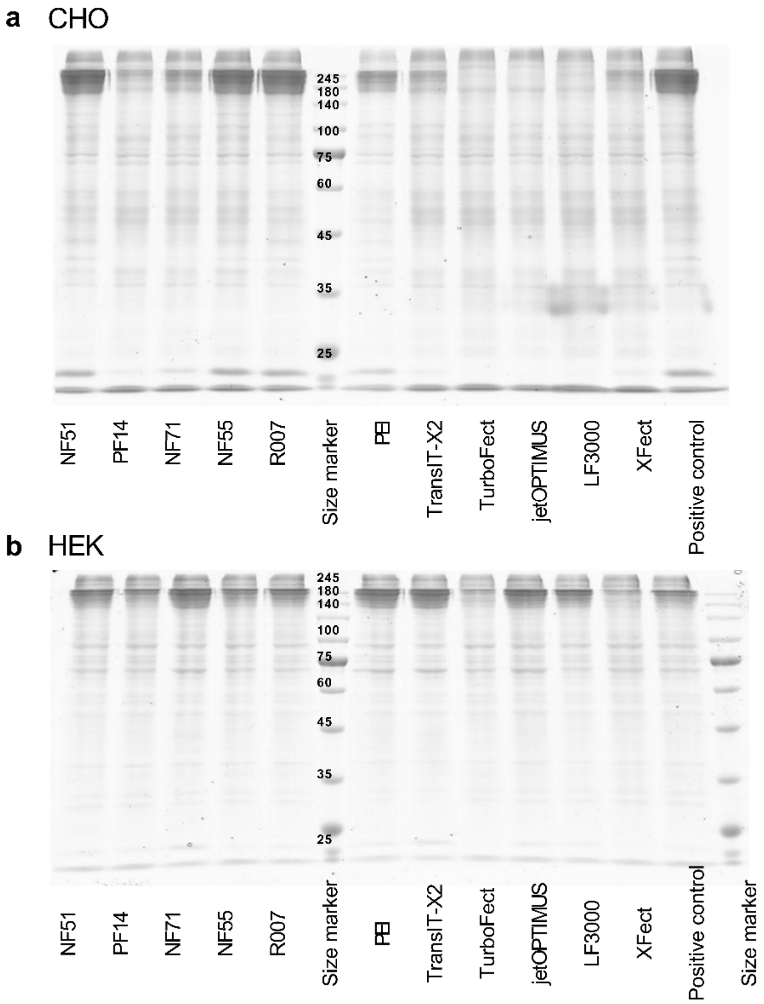

Figure S14. Full SDS gel images used in Figure 3. CHO and HEK cells used to express Trastuzumab mAB in suspension cell culture. 2 µg of pLic2.1 pDNA per 6-well plate well was used. For CHO 1E9 (a) cells transfection in Xell CHO TF serum free media was used and analysis was performed 8 days post-transfection. For HEK293ALL(b) BalanCD HEK293 serum free media was used and analysis was performed 5 days post-transfection. For CPP/pDNA complexes CR2.5 was used. Positive control - used during production to be sure the production works.

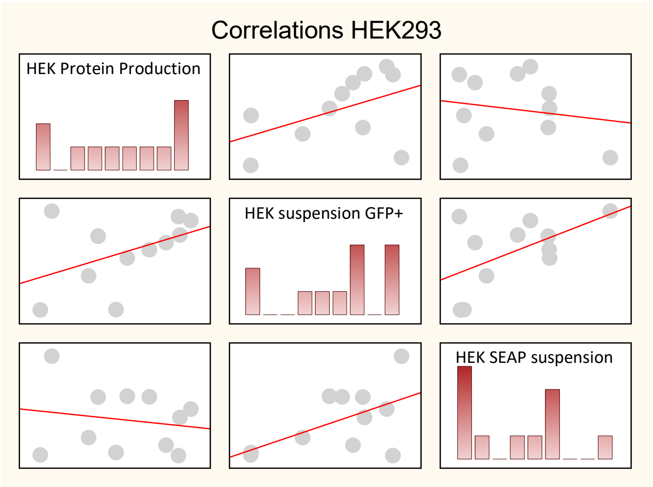

Figure S15. Scatterplot that highlights the correlations between the protein production yields and the assays of transfection-positive cell population and SEAP protein secretion in HEK293 cells.

Table S1. Cell penetrating peptides used in this work, and their sequences

Name	Abbreviation	Sequence	Reference
NickFect51	NF51	Stearoyl-AGYLLGO[a]INLKALAALAKKIL[b]	Arukuusk, et al, 2013, [8]
NickFect71	NF71	Stearoyl-HHYHHGO[a]ILLKALKALAKAIL[b]	Porosk, et al, 2019, [11]
PepFect14	PF14	Stearoyl-AGYLLGKLLOOLAAAALOOLL[b]	Veiman, et al 2013, [12]
NickFect55	NF55	Stearoyl-AGYLLGO[a]INLKALAALAKAIL[b]	Freimann, et al 2016, [9]

a – The synthesis is continued from the sidechain amino group of the amino acid ornithine, instead of alpha-amino group as usually.
b – The peptides are C-terminally amidated.

Table S2. Statistical analysis of the transfection efficacy
Total reporter from the cell lysate in CHO adherent culture.
Statistical analysis of the variance (1-way ANOVA) for the dataset presented in Figure 1A is presented below, together with Tukey post-hoc test p values.

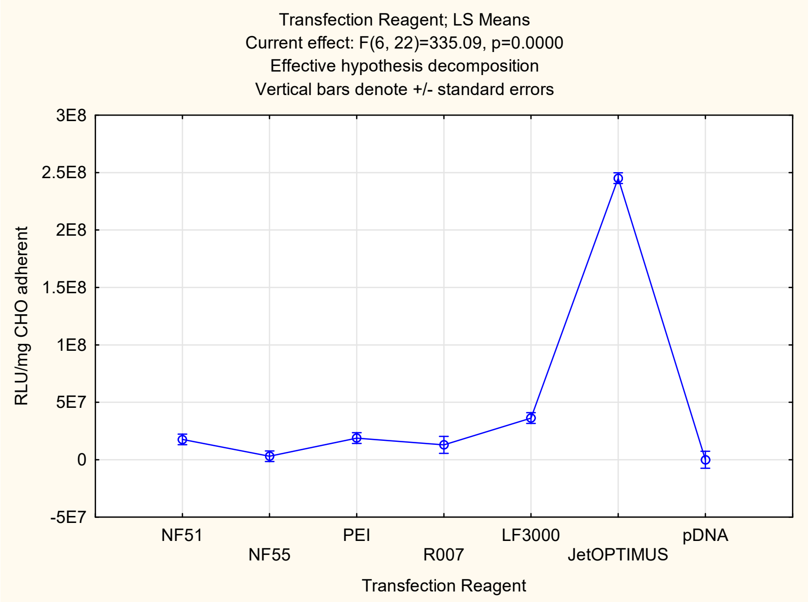

Tukey HSD test; variable RLU/mg CHO adherent
Approximate Probabilities for Post Hoc Tests
Error: Between MS = 109 ? 10^{12}, df = 22.000, Red highlight: p<0.05

Cell No.	Transfection Reagent	{1} 1766E4	{2} 3050E3	{3} 1878E4	{4} 1291E4	{5} 3626E4	{6} 2452E5	{7} 1715.0
1	NF51		0.329	1.000	0.998	0.116	0.000	0.430
2	NF55	0.329		0.252	0.912	0.001	0.000	1.000
3	PEI	1.000	0.252		0.993	0.160	0.000	0.360
4	R007	0.998	0.912	0.993		0.153	0.000	0.872
5	LF3000	0.116	0.001	0.160	0.153		0.000	0.007
6	JetOPTIMUS	0.000	0.000	0.000	0.000	0.000		0.000
7	pDNA	0.430	1.000	0.360	0.872	0.007	0.000	

Statistical analysis of the transfection efficacy
Total reporter from the cell lysate in CHO adherent culture. Extended set of transfection reagents.
Statistical analysis of the variance (1-way ANOVA) for the dataset presented in Figure 2A is presented below, together with Tukey post-hoc test p values.

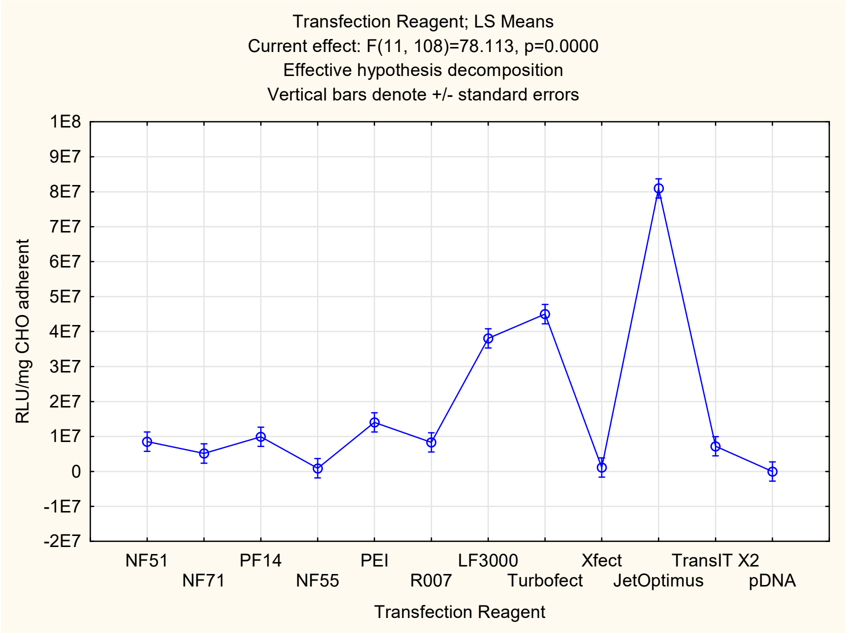

Tukey HSD test; variable RLU/mg CHO adherent
Approximate Probabilities for Post Hoc Tests
Error: Between MS = 761 x 10^{11}, df = 108.00, Red highlight: p<0.05

Cell No.	Transfection Reagent	{1} 8528E3	{2} 5153E3	{3} 9915E3	{4} 9014E2	{5} 1403E4	{6} 8316E3	{7} 3805E4	{8} 4501E4	{9} 1138E3	{10} 8095E4	{11} 7215E3	{12} 239.93
1	NF51		0.999	1.000	0.722	0.959	1.000	0.000	0.000	0.760	0.000	1.000	0.564
2	NF71	0.999		0.986	0.995	0.500	1.000	0.000	0.000	0.997	0.000	1.000	0.975
3	PF14	1.000	0.986		0.476	0.996	1.000	0.000	0.000	0.518	0.000	1.000	0.327
4	NF55	0.722	0.995	0.476		0.047	0.757	0.000	0.000	1.000	0.000	0.899	1.000
5	PEI	0.959	0.500	0.996	0.047		0.947	0.000	0.000	0.055	0.000	0.842	0.024
6	R007	1.000	1.000	1.000	0.757	0.947		0.000	0.000	0.792	0.000	1.000	0.602
7	LF3000	0.000	0.000	0.000	0.000	0.000	0.000		0.824	0.000	0.000	0.000	0.000
8	Turbofect	0.000	0.000	0.000	0.000	0.000	0.000	0.824		0.000	0.000	0.000	0.000
9	Xfect	0.760	0.997	0.518	1.000	0.055	0.792	0.000	0.000		0.000	0.920	1.000
10	JetOptimus	0.000	0.000	0.000	0.000	0.000	0.000	0.000	0.000	0.000		0.000	0.000
11	TransIT X2	1.000	1.000	1.000	0.899	0.842	1.000	0.000	0.000	0.920	0.000		0.787
12	pDNA	0.564	0.975	0.327	1.000	0.024	0.602	0.000	0.000	1.000	0.000	0.787	

Statistical analysis of the transfection efficacy

Total reporter from the cell lysate in HEK293 adherent culture.

Statistical analysis of the variance (1-way ANOVA) for the dataset presented in Figure 2A is presented below, together with Tukey post-hoc test p values.

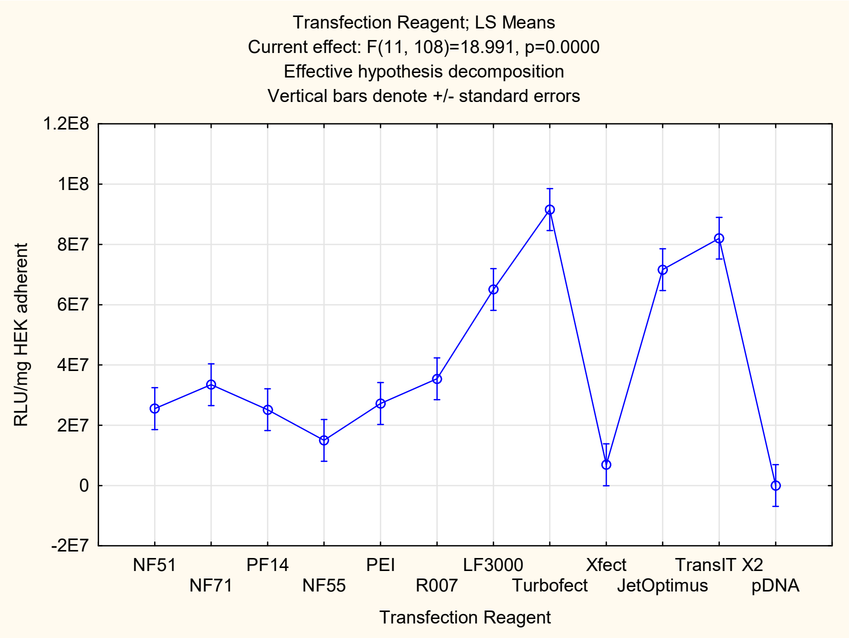

Tukey HSD test; variable RLU/mg CHO adherent
Approximate Probabilities for Post Hoc Tests
Error: Between MS = 480 ? 10^{12}, df = 108.00, Red highlight: $p<0.05$

Cell No.	Transfection Reagent	{1} 2552E4	{2} 3347E4	{3} 2517E4	{4} 1497E4	{5} 2723E4	{6} 3543E4	{7} 6505E4	{8} 9157E4	{9} 6876E3	{10} 7166E4	{11} 8205E4	{12} 306.80
1	NF51		1.000	1.000	0.995	1.000	0.997	0.006	0.000	0.756	0.001	0.000	0.291
2	NF71	1.000		0.999	0.764	1.000	1.000	0.070	0.000	0.234	0.009	0.000	0.041
3	PF14	1.000	0.999		0.996	1.000	0.996	0.005	0.000	0.777	0.001	0.000	0.312
4	NF55	0.995	0.764	0.996		0.983	0.633	0.000	0.000	1.000	0.000	0.000	0.930
5	PEI	1.000	1.000	1.000	0.983		1.000	0.010	0.000	0.641	0.001	0.000	0.205
6	R007	0.997	1.000	0.996	0.633	1.000		0.117	0.000	0.151	0.017	0.000	0.022
7	LF3000	0.006	0.070	0.005	0.000	0.010	0.117		0.238	0.000	1.000	0.848	0.000
8	Turbofect	0.000	0.000	0.000	0.000	0.000	0.000	0.238		0.000	0.671	0.998	0.000
9	Xfect	0.756	0.234	0.777	1.000	0.641	0.151	0.000	0.000		0.000	0.000	1.000
10	JetOptimus	0.001	0.009	0.001	0.000	0.001	0.017	1.000	0.671	0.000		0.996	0.000
11	TransIT X2	0.000	0.000	0.000	0.000	0.000	0.000	0.848	0.998	0.000	0.996		0.000
12	pDNA	0.291	0.041	0.312	0.930	0.205	0.022	0.000	0.000	1.000	0.000	0.000	

Statistical analysis of the transfection efficacy

Total reporter from the cell lysate in CHO suspension culture.

Statistical analysis of the variance (1-way ANOVA) for the dataset presented in Figure 2B is presented below, together with Tukey post-hoc test p values.

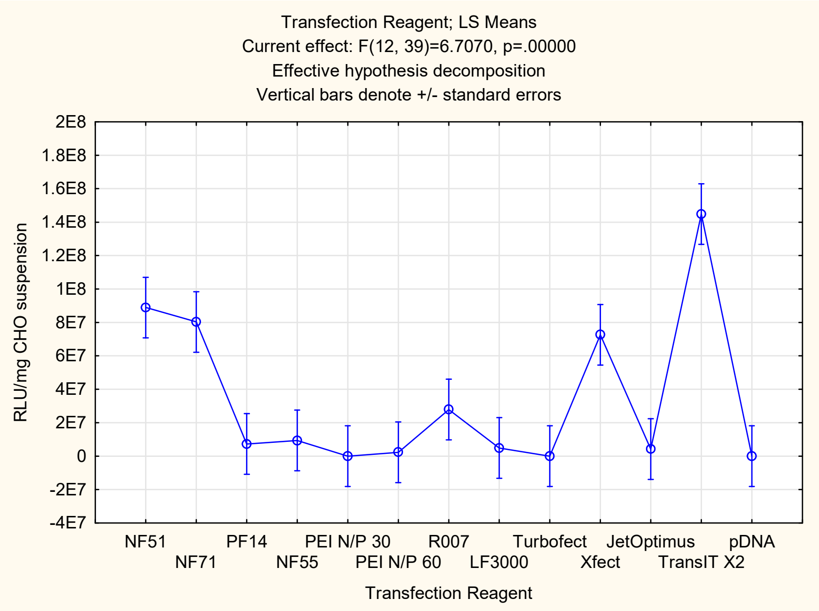

Tukey HSD test; variable RLU/mg suspension
Approximate Probabilities for Post Hoc Tests
Error: Between MS = 132 ? 10^{13}, df = 39.000, Red highlight: p<0.05

Cell No.	Transfection Reagent	{1} 8890E4	{2} 8029E4	{3} 7285E3	{4} 9392E3	{5} 3411.7	{6} 2304E3	{7} 2788E4	{8} 4898E3	{9} 2644.1	{10} 7261E4	{11} 4183E3	{12} 1448E5	{13} 1038.3
1	NF51		1.000	0.112	0.134	0.058	0.072	0.479	0.091	0.058	1.000	0.085	0.610	0.058
2	NF71	1.000		0.223	0.260	0.125	0.152	0.700	0.186	0.125	1.000	0.176	0.393	0.125
3	PF14	0.112	0.223		1.000	1.000	1.000	1.000	1.000	1.000	0.375	1.000	0.000	1.000
4	NF55	0.134	0.260	1.000		1.000	1.000	1.000	1.000	1.000	0.425	1.000	0.000	1.000
5	PEI N/P 30	0.058	0.125	1.000	1.000		1.000	0.996	1.000	1.000	0.230	1.000	0.000	1.000
6	PEI N/P 60	0.072	0.152	1.000	1.000	1.000		0.998	1.000	1.000	0.271	1.000	0.000	1.000
7	R007	0.479	0.700	1.000	1.000	0.996	0.998		0.999	0.996	0.865	0.999	0.003	0.996
8	LF3000	0.091	0.186	1.000	1.000	1.000	1.000	0.999		1.000	0.323	1.000	0.000	1.000
9	Turbofect	0.058	0.125	1.000	1.000	1.000	1.000	0.996	1.000		0.230	1.000	0.000	1.000
10	Xfect	1.000	1.000	0.375	0.425	0.230	0.271	0.865	0.323	0.230		0.308	0.236	0.230
11	JetOptimus	0.085	0.176	1.000	1.000	1.000	1.000	0.999	1.000	1.000	0.308		0.000	1.000
12	TransIT X2	0.610	0.393	0.000	0.000	0.000	0.000	0.003	0.000	0.000	0.236	0.000		0.000
13	pDNA	0.058	0.125	1.000	1.000	1.000	1.000	0.996	1.000	1.000	0.230	1.000	0.000	

Statistical analysis of the transfection efficacy

Total reporter from the cell lysate in HEK293 suspension culture.

Statistical analysis of the variance (1-way ANOVA) for the dataset presented in Figure 2B is presented below, together with Tukey post-hoc test p values.

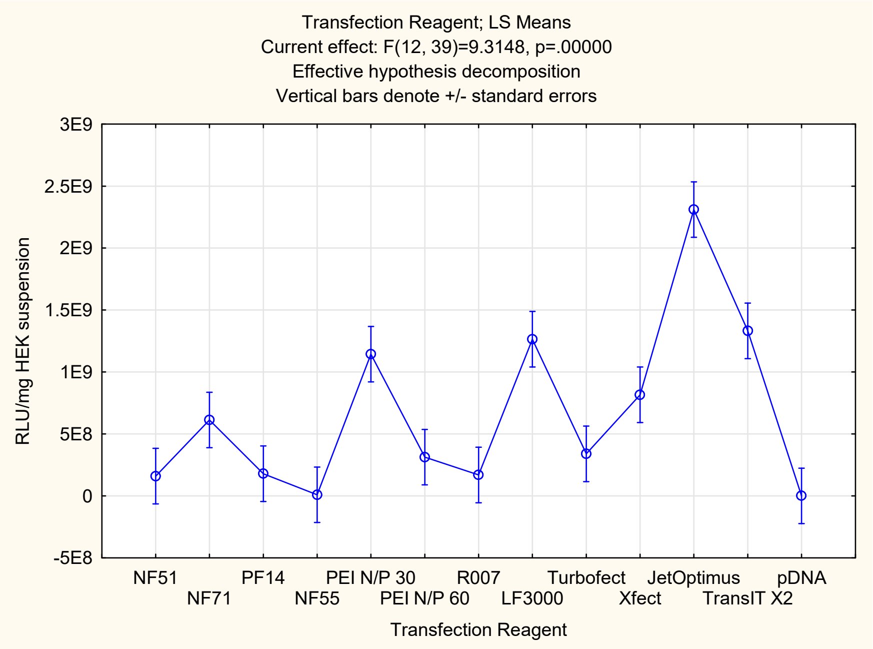

Tukey HSD test; variable RLU/mg suspension
Approximate Probabilities for Post Hoc Tests
Error: Between MS = 201 ? 10^{15}, df = 39.000, Red highlight: $p<0.05$

Cell No.	Transfection Reagent	{1} 1594E5	{2} 6124E5	{3} 1793E5	{4} 9611E3	{5} 1143E6	{6} 3125E5	{7} 1691E5	{8} 1264E6	{9} 3392E5	{10} 8156E5	{11} 2310E6	{12} 1332E6	{13} 25668.
1	NF51		0.963	1.000	1.000	0.132	1.000	1.000	0.055	1.000	0.681	0.000	0.032	1.000
2	NF71	0.963		0.973	0.783	0.892	0.999	0.968	0.690	1.000	1.000	0.000	0.550	0.766
3	PF14	1.000	0.973		1.000	0.150	1.000	1.000	0.064	1.000	0.721	0.000	0.038	1.000
4	NF55	1.000	0.783	1.000		0.044	0.999	1.000	0.016	0.997	0.375	0.000	0.009	1.000
5	PEI N/P 30	0.132	0.892	0.150	0.044		0.331	0.140	1.000	0.379	0.997	0.033	1.000	0.040
6	PEI N/P 60	1.000	0.999	1.000	0.999	0.331		1.000	0.163	1.000	0.923	0.000	0.103	0.998
7	R007	1.000	0.968	1.000	1.000	0.140	1.000		0.059	1.000	0.700	0.000	0.035	1.000
8	LF3000	0.055	0.690	0.064	0.016	1.000	0.163	0.059		0.193	0.965	0.085	1.000	0.015
9	Turbofect	1.000	1.000	1.000	0.997	0.379	1.000	1.000	0.193		0.946	0.000	0.124	0.997
10	Xfect	0.681	1.000	0.721	0.375	0.997	0.923	0.700	0.965	0.946		0.002	0.910	0.358
11	JetOptimus	0.000	0.000	0.000	0.000	0.033	0.000	0.000	0.085	0.000	0.002		0.136	0.000
12	TransIT X2	0.032	0.550	0.038	0.009	1.000	0.103	0.035	1.000	0.124	0.910	0.136		0.008
13	pDNA	1.000	0.766	1.000	1.000	0.040	0.998	1.000	0.015	0.997	0.358	0.000	0.008	

Statistical analysis of the transfection efficacy

Transfection-positive cell population in CHO adherent culture.

Statistical analysis of the variance (1-way ANOVA) for the dataset presented in Figure 2C is presented below, together with Tukey post-hoc test p values.

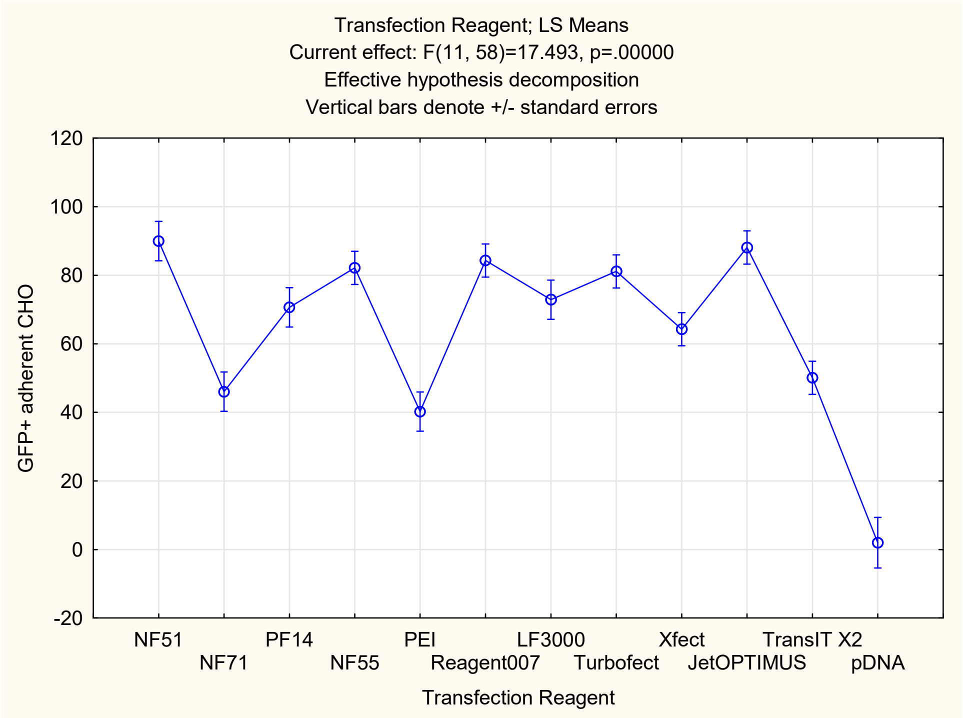

Tukey HSD test; variable GFP+ adherent
Approximate Probabilities for Post Hoc Tests
Error: Between MS = 163.95, df = 58.000, Red highlight: p<0.05

Cell No.	Transfection Reagent	{1} 89.958	{2} 46.046	{3} 70.645	{4} 82.163	{5} 40.245	{6} 84.318	{7} 72.867	{8} 81.137	{9} 64.249	{10} 88.089	{11} 50.068	{12} 1.9890
1	NF51		0.000	0.432	0.996	0.000	1.000	0.618	0.989	0.047	1.000	0.000	0.000
2	NF71	0.000		0.124	0.001	1.000	0.000	0.064	0.001	0.405	0.000	1.000	0.001
3	PF14	0.432	0.124		0.924	0.019	0.798	1.000	0.959	0.999	0.471	0.232	0.000
4	NF55	0.996	0.001	0.924		0.000	1.000	0.983	1.000	0.294	0.999	0.001	0.000
5	PEI	0.000	1.000	0.019	0.000		0.000	0.008	0.000	0.084	0.000	0.974	0.007
6	Reagent007	1.000	0.000	0.798	1.000	0.000		0.927	1.000	0.157	1.000	0.000	0.000
7	LF3000	0.618	0.064	1.000	0.983	0.008	0.927		0.993	0.991	0.672	0.123	0.000
8	Turbofect	0.989	0.001	0.959	1.000	0.000	1.000	0.993		0.380	0.997	0.002	0.000
9	Xfect	0.047	0.405	0.999	0.294	0.084	0.157	0.991	0.380		0.041	0.644	0.000
10	JetOPTIMUS	1.000	0.000	0.471	0.999	0.000	1.000	0.672	0.997	0.041		0.000	0.000
11	TransIT X2	0.000	1.000	0.232	0.001	0.974	0.000	0.123	0.002	0.644	0.000		0.000
12	pDNA	0.000	0.001	0.000	0.000	0.007	0.000	0.000	0.000	0.000	0.000	0.000	

Statistical analysis of the transfection efficacy

Transfection-positive cell population in HEK293 adherent culture.

Statistical analysis of the variance (1-way ANOVA) for the dataset presented in Figure 2C is presented below, together with Tukey post-hoc test p values.

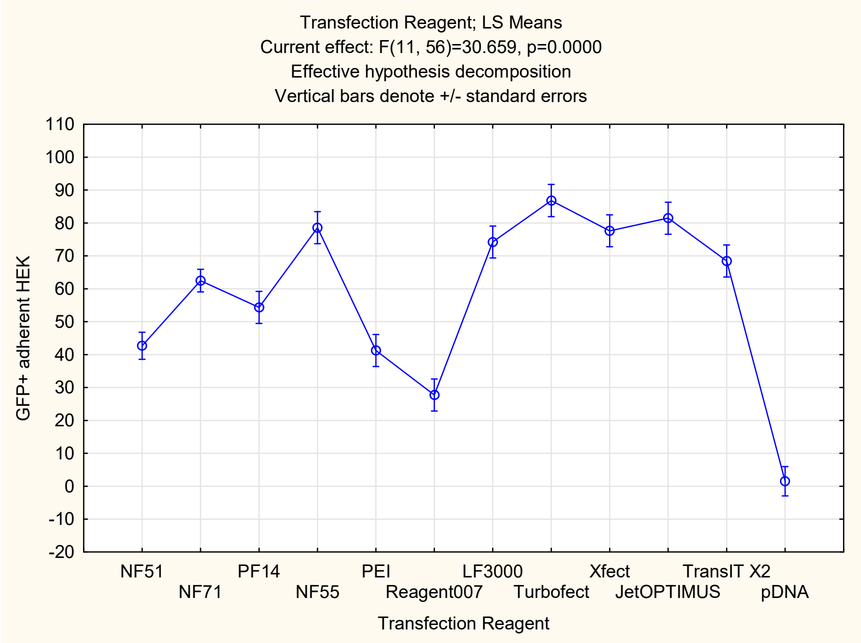

Tukey HSD test; variable GFP+ adherent
Approximate Probabilities for Post Hoc Tests
Error: Between MS = 118.10, df = 56.000, Red highlight: p<0.05

Cell No.	Transfection Reagent	{1} 42.670	{2} 62.481	{3} 54.338	{4} 78.594	{5} 41.244	{6} 27.726	{7} 74.236	{8} 86.836	{9} 77.639	{10} 81.480	{11} 68.457	{12} 1.5035
1	NF51		0.023	0.793	0.000	1.000	0.457	0.001	0.000	0.000	0.000	0.008	0.000
2	NF71	0.023		0.965	0.250	0.033	0.000	0.708	0.007	0.334	0.087	0.997	0.000
3	PF14	0.793	0.965		0.036	0.751	0.014	0.171	0.001	0.053	0.011	0.656	0.000
4	NF55	0.000	0.250	0.036		0.000	0.000	1.000	0.987	1.000	1.000	0.941	0.000
5	PEI	1.000	0.033	0.751	0.000		0.713	0.001	0.000	0.000	0.000	0.011	0.000
6	Reagent007	0.457	0.000	0.014	0.000	0.713		0.000	0.000	0.000	0.000	0.000	0.010
7	LF3000	0.001	0.708	0.171	1.000	0.001	0.000		0.793	1.000	0.995	0.999	0.000
8	Turbofect	0.000	0.007	0.001	0.987	0.000	0.000	0.793		0.970	1.000	0.266	0.000
9	Xfect	0.000	0.334	0.053	1.000	0.000	0.000	1.000	0.970		1.000	0.970	0.000
10	JetOPTIMUS	0.000	0.087	0.011	1.000	0.000	0.000	0.995	1.000	1.000		0.757	0.000
11	TransIT X2	0.008	0.997	0.656	0.941	0.011	0.000	0.999	0.266	0.970	0.757		0.000
12	pDNA	0.000	0.000	0.000	0.000	0.000	0.010	0.000	0.000	0.000	0.000	0.000	

Statistical analysis of the transfection efficacy

Transfection-positive cell population in CHO suspension culture.

Statistical analysis of the variance (1-way ANOVA) for the dataset presented in Figure 2D is presented below, together with Tukey post-hoc test p values.

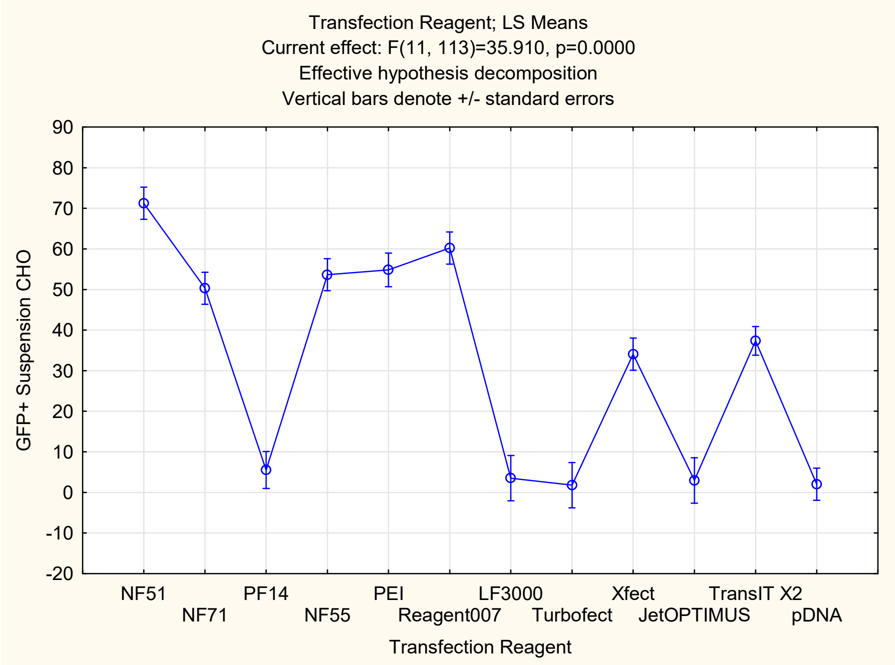

Tukey HSD test; variable GFP+ Suspension
Approximate Probabilities for Post Hoc Tests
Error: Between MS = 187.87, df = 113.00, Red highlight: p<0.05

Cell No.	Transfection Reagent	{1} 71.247	{2} 50.300	{3} 5.5441	{4} 53.650	{5} 54.825	{6} 60.216	{7} 3.5202	{8} 1.7970	{9} 34.047	{10} 2.9652	{11} 37.342	{12} 2.0138
1	NF51		0.015	0.000	0.085	0.166	0.711	0.000	0.000	0.000	0.000	0.000	0.000
2	NF71	0.015		0.000	1.000	1.000	0.829	0.000	0.000	0.154	0.000	0.388	0.000
3	PF14	0.000	0.000		0.000	0.000	0.000	1.000	1.000	0.001	1.000	0.000	1.000
4	NF55	0.085	1.000	0.000		1.000	0.990	0.000	0.000	0.031	0.000	0.102	0.000
5	PEI	0.166	1.000	0.000	1.000		0.999	0.000	0.000	0.021	0.000	0.071	0.000
6	Reagent007	0.711	0.829	0.000	0.990	0.999		0.000	0.000	0.001	0.000	0.002	0.000
7	LF3000	0.000	0.000	1.000	0.000	0.000	0.000		1.000	0.001	1.000	0.000	1.000
8	Turbofect	0.000	0.000	1.000	0.000	0.000	0.000	1.000		0.001	1.000	0.000	1.000
9	Xfect	0.000	0.154	0.001	0.031	0.021	0.001	0.001	0.001		0.001	1.000	0.000
10	JetOPTIMUS	0.000	0.000	1.000	0.000	0.000	0.000	1.000	1.000	0.001		0.000	1.000
11	TransIT X2	0.000	0.388	0.000	0.102	0.071	0.002	0.000	0.000	1.000	0.000		0.000
12	pDNA	0.000	0.000	1.000	0.000	0.000	0.000	1.000	1.000	0.000	1.000	0.000	

Statistical analysis of the transfection efficacy

Transfection-positive cell population in HEK293 suspension culture.

Statistical analysis of the variance (1-way ANOVA) for the dataset presented in Figure 2D is presented below, together with Tukey post-hoc test p values.

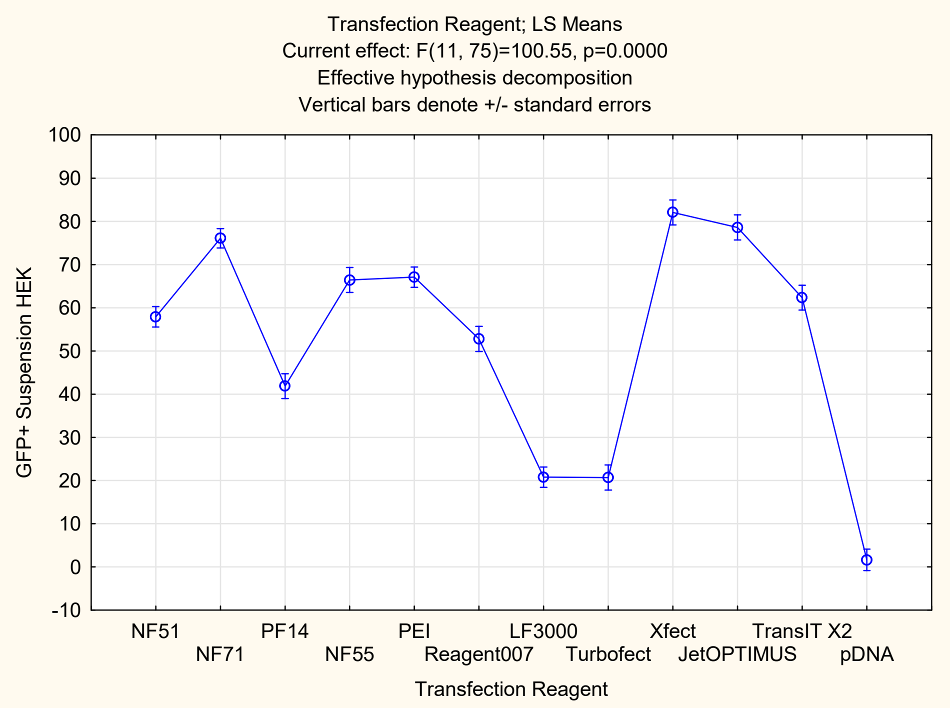

Tukey HSD test; variable GFP+ Suspension
Approximate Probabilities for Post Hoc Tests
Error: Between MS = 49.947, df = 75.000, Red highlight: p<0.05

Cell No.	Transfection Reagent	{1} 57.922	{2} 76.076	{3} 41.856	{4} 66.428	{5} 67.071	{6} 52.777	{7} 20.779	{8} 20.710	{9} 82.072	{10} 78.592	{11} 62.335	{12} 1.6703
1	NF51		0.000	0.003	0.497	0.225	0.964	0.000	0.000	0.000	0.000	0.989	0.000
2	NF71	0.000		0.000	0.275	0.213	0.000	0.000	0.000	0.887	1.000	0.016	0.000
3	PF14	0.003	0.000		0.000	0.000	0.259	0.000	0.000	0.000	0.000	0.000	0.000
4	NF55	0.497	0.275	0.000		1.000	0.054	0.000	0.000	0.013	0.135	0.997	0.000
5	PEI	0.225	0.213	0.000	1.000		0.013	0.000	0.000	0.007	0.103	0.980	0.000
6	Reagent007	0.964	0.000	0.259	0.054	0.013		0.000	0.000	0.000	0.000	0.457	0.000
7	LF3000	0.000	0.000	0.000	0.000	0.000	0.000		1.000	0.000	0.000	0.000	0.000
8	Turbofect	0.000	0.000	0.000	0.000	0.000	0.000	1.000		0.000	0.000	0.000	0.000
9	Xfect	0.000	0.887	0.000	0.013	0.007	0.000	0.000	0.000		0.999	0.001	0.000
10	JetOPTIMUS	0.000	1.000	0.000	0.135	0.103	0.000	0.000	0.000	0.999		0.008	0.000
11	TransIT X2	0.989	0.016	0.000	0.997	0.980	0.457	0.000	0.000	0.001	0.008		0.000
12	pDNA	0.000	0.000	0.000	0.000	0.000	0.000	0.000	0.000	0.000	0.000	0.000	

Statistical analysis of the transfection efficacy

Total secreted protein in CHO suspension culture.

Statistical analysis of the variance (1-way ANOVA) for the dataset presented in Figure 3A is presented below, together with Tukey post-hoc test p values.

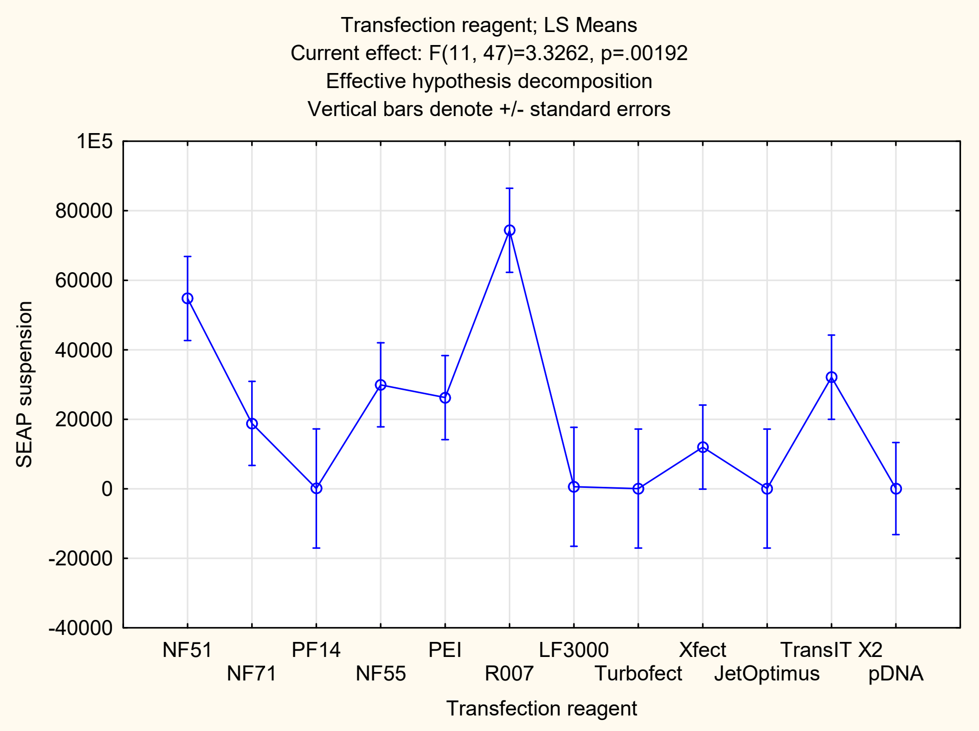

Tukey HSD test; variable SEAP suspension
Approximate Probabilities for Post Hoc Tests
Error: Between MS = 8783 ? 10^5, df = 47.000, Red highlight: p<0.05

Cell No.	Transfection reagent	{1} 54775.	{2} 18828.	{3} 104.00	{4} 29900.	{5} 26256.	{6} 74385.	{7} 591.00	{8} 48.333	{9} 11978.	{10} 68.667	{11} 32092.	{12} 67.200
1	NF51		0.625	0.304	0.946	0.874	0.991	0.317	0.303	0.364	0.303	0.971	0.127
2	NF71	0.625		0.999	1.000	1.000	0.080	0.999	0.999	1.000	0.999	1.000	0.996
3	PF14	0.304	0.999		0.953	0.982	0.038	1.000	1.000	1.000	1.000	0.925	1.000
4	NF55	0.946	1.000	0.953		1.000	0.309	0.958	0.953	0.996	0.953	1.000	0.876
5	PEI	0.874	1.000	0.982	1.000		0.208	0.984	0.981	0.999	0.981	1.000	0.944
6	R007	0.991	0.080	0.038	0.309	0.208		0.040	0.038	0.029	0.038	0.382	0.007
7	LF3000	0.317	0.999	1.000	0.958	0.984	0.040		1.000	1.000	1.000	0.932	1.000
8	Turbofect	0.303	0.999	1.000	0.953	0.981	0.038	1.000		1.000	1.000	0.925	1.000
9	Xfect	0.364	1.000	1.000	0.996	0.999	0.029	1.000	1.000		1.000	0.988	1.000
10	JetOptimus	0.303	0.999	1.000	0.953	0.981	0.038	1.000	1.000	1.000		0.925	1.000
11	TransIT X2	0.971	1.000	0.925	1.000	1.000	0.382	0.932	0.925	0.988	0.925		0.818
12	pDNA	0.127	0.996	1.000	0.876	0.944	0.007	1.000	1.000	1.000	1.000	0.818	

Statistical analysis of the transfection efficacy

Total secreted protein in HEK293 suspension culture.

Statistical analysis of the variance (1-way ANOVA) for the dataset presented in Figure 3A is presented below, together with Tukey post-hoc test p values.

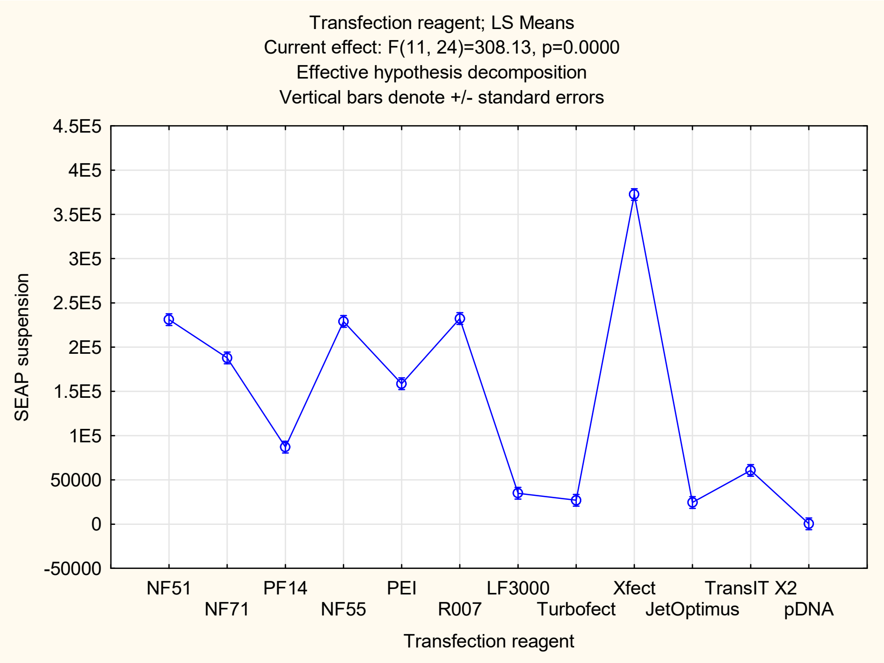

Tukey HSD test; variable SEAP suspension
Approximate Probabilities for Post Hoc Tests
Error: Between MS = 1302 ? 10^5, df = 24.000, Red highlight: p<0.05

Cell No.	Transfection reagent	{1} 2311E	{2} 1879E	{3} 87038.	{4} 2291E	{5} 1586E	{6} 2323E	{7} 34843.	{8} 26884.	{9} 3725E	{10} 24405.	{11} 60608.	{12} 433.33
1	NF51		0.005	0.000	1.000	0.000	1.000	0.000	0.000	0.000	0.000	0.000	0.000
2	NF71	0.005		0.000	0.008	0.131	0.004	0.000	0.000	0.000	0.000	0.000	0.000
3	PF14	0.000	0.000		0.000	0.000	0.000	0.001	0.000	0.000	0.000	0.226	0.000
4	NF55	1.000	0.008	0.000		0.000	1.000	0.000	0.000	0.000	0.000	0.000	0.000
5	PEI	0.000	0.131	0.000	0.000		0.000	0.000	0.000	0.000	0.000	0.000	0.000
6	R007	1.000	0.004	0.000	1.000	0.000		0.000	0.000	0.000	0.000	0.000	0.000
7	LF3000	0.000	0.000	0.001	0.000	0.000	0.000		0.999	0.000	0.990	0.255	0.041
8	Turbofect	0.000	0.000	0.000	0.000	0.000	0.000	0.999		0.000	1.000	0.049	0.225
9	Xfect	0.000	0.000	0.000	0.000	0.000	0.000	0.000	0.000		0.000	0.000	0.000
10	JetOptimus	0.000	0.000	0.000	0.000	0.000	0.000	0.990	1.000	0.000		0.027	0.346
11	TransIT X2	0.000	0.000	0.226	0.000	0.000	0.000	0.255	0.049	0.000	0.027		0.000
12	pDNA	0.000	0.000	0.000	0.000	0.000	0.000	0.041	0.225	0.000	0.346	0.000	

Supplementary Table 3

Statistical analysis of the transfection efficacy

Correlation matrixes between the numerical outputs of the various transfection assays, toxicity assays, and protein yields for both CHO and HEK293 cell lines. Pearson correlation are shown and significant correlations (p<.05) are highlighted in red.

Correlation matrixes: CHO

Correlations with means
Marked correlations are significant at $p < 0.05000$
N=11 (Casewise deletion of missing data)

Variable	Means	Std.Dev.	CHO adherent Luc	CHO suspension Luc	CHO SEAP suspension	CHO adherent GFP+	CHO suspension GFP+	CHO suspension GFP_low	CHO suspension GFP_med	CHO suspension GFP_high	CHO Protein Production	CHO Adherent Live	CHO Adherent Dead	CHO suspension Live	CHO suspension Dead	CHO adhesion MTS	CHO adhesion BrdU
CHO adherent Luc	19928765	24852593	1.00	-0.47	-0.51	0.25	-0.69	-0.73	-0.64	-0.60	-0.59	-0.85	0.76	0.06	-0.12	-0.44	-0.31
CHO suspension Luc	40235670	48744235	-0.47	1.00	0.36	0.16	0.43	0.40	0.28	0.39	0.18	0.16	-0.04	0.07	-0.09	0.33	-0.41
CHO SEAP suspension	22639	24510	-0.51	0.36	1.00	0.33	0.85	0.69	0.80	0.93	0.88	0.53	-0.40	-0.04	0.06	-0.22	-0.06
CHO adherent GFP+	71	14	0.25	0.16	0.33	1.00	0.21	0.21	0.30	0.23	0.34	-0.31	0.17	-0.20	0.08	-0.34	-0.01
CHO suspension GFP+	34	26	-0.69	0.43	0.85	0.21	1.00	0.95	0.97	0.97	0.86	0.60	-0.46	0.10	-0.09	-0.19	0.16
CHO suspension GFP_low	13	9	-0.73	0.40	0.69	0.21	0.95	1.00	0.96	0.87	0.78	0.57	-0.45	0.11	-0.12	-0.11	0.31
CHO suspension GFP_med	12	11	-0.64	0.28	0.80	0.30	0.97	0.96	1.00	0.94	0.89	0.58	-0.50	0.09	-0.09	-0.23	0.35
CHO suspension GFP_high	9	8	-0.60	0.39	0.93	0.23	0.97	0.87	0.94	1.00	0.86	0.55	-0.42	0.12	-0.11	-0.25	0.10
CHO Protein Production	65	34	-0.59	0.18	0.88	0.34	0.86	0.78	0.89	0.86	1.00	0.62	-0.53	-0.09	0.12	-0.29	0.21
CHO Adherent Live	73	17	-0.85	0.16	0.53	-0.31	0.60	0.57	0.58	0.55	0.62	1.00	-0.91	-0.27	0.33	0.43	0.36
CHO Adherent Dead	6	5	0.76	-0.04	-0.40	0.17	-0.46	-0.45	-0.50	-0.42	-0.53	-0.91	1.00	0.41	-0.45	-0.51	-0.55
CHO suspension Live	60	9	0.06	0.07	-0.04	-0.20	0.10	0.11	0.09	0.12	-0.09	-0.27	0.41	1.00	-0.98	-0.34	0.10
CHO suspension Dead	30	8	-0.12	-0.09	0.06	0.08	-0.09	-0.12	-0.09	-0.11	0.12	0.33	-0.45	-0.98	1.00	0.32	-0.11
CHO adhesion MTS	59	26	-0.44	0.33	-0.22	-0.34	-0.19	-0.11	-0.23	-0.25	-0.29	0.43	-0.51	-0.34	0.32	1.00	0.14
CHO adhesion BrdU	1	0	-0.31	-0.41	-0.06	-0.01	0.16	0.31	0.35	0.10	0.21	0.36	-0.55	0.10	-0.11	0.14	1.00

Correlation matrixes: HEK293

Correlations with means)
Marked correlations are significant at $p < 0.05000$
N=11 (Casewise deletion of missing data)

Variable	Means	Std.Dev.	HEK adherent Luc	HEK suspension Luc	HEK SEAP suspension	HEK adherent GFP +	HEK suspension GFP+	HEK suspension GFP_low	HEK suspension GFP_med	HEK suspension GFP_high	HEK Protein Production	HEK adherent Live	HEK adherent Dead	HEK suspension Live	HEK suspension Dead	HEK adhesion MTS	HEK adhesion BrdU
HEK adherent Luc	43544619	28808114	1.00	0.48	-0.84	0.41	-0.48	-0.20	-0.53	-0.42	0.06	0.19	-0.25	-0.17	0.11	-0.48	0.01
HEK suspension Luc	########	########	0.48	1.00	-0.46	0.34	0.26	-0.44	-0.08	0.41	0.47	-0.05	0.12	-0.01	0.00	-0.38	-0.50
HEK SEAP suspension	149568	112591	-0.84	-0.46	1.00	-0.31	0.55	0.09	0.42	0.52	-0.17	0.17	-0.06	0.19	-0.11	0.51	-0.04
HEK adherent GFP+	63	19	0.41	0.34	-0.31	1.00	-0.07	-0.18	-0.42	-0.09	-0.39	0.05	-0.11	0.18	-0.17	-0.09	-0.51
HEK suspension GFP+	57	21	-0.48	0.26	0.55	-0.07	1.00	-0.02	0.72	0.88	0.46	-0.29	0.47	-0.05	0.18	0.13	-0.13
HEK suspension GFP_low	17	8	-0.20	-0.44	0.09	-0.18	-0.02	1.00	0.16	-0.44	0.13	-0.38	0.21	-0.78	0.80	-0.27	0.58
HEK suspension GFP_med	11	4	-0.53	-0.08	0.42	-0.42	0.72	0.16	1.00	0.57	0.67	-0.40	0.54	-0.24	0.28	0.10	0.12
HEK suspension GFP_high	34	21	-0.42	0.41	0.52	-0.09	0.88	-0.44	0.57	1.00	0.38	-0.03	0.26	0.27	-0.20	0.19	-0.38
HEK Protein Production	64	16	0.06	0.47	-0.17	-0.39	0.46	0.13	0.67	0.38	1.00	-0.36	0.44	-0.54	0.49	-0.44	0.11
HEK adherent Live	79	7	0.19	-0.05	0.17	0.05	-0.29	-0.38	-0.40	-0.03	-0.36	1.00	-0.96	0.17	-0.24	0.38	-0.09
HEK adherent Dead	7	3	-0.25	0.12	-0.06	-0.11	0.47	0.21	0.54	0.26	0.44	-0.96	1.00	-0.05	0.13	-0.31	0.02
HEK suspension Live	62	12	-0.17	-0.01	0.19	0.18	-0.05	-0.78	-0.24	0.27	-0.54	0.17	-0.05	1.00	-0.97	0.39	-0.55
HEK suspension Dead	23	11	0.11	0.00	-0.11	-0.17	0.18	0.80	0.28	-0.20	0.49	-0.24	0.13	-0.97	1.00	-0.28	0.59
HEK adhesion MTS	50	24	-0.48	-0.38	0.51	-0.09	0.13	-0.27	0.10	0.19	-0.44	0.38	-0.31	0.39	-0.28	1.00	-0.04
HEK adhesion BrdU	1	0	0.01	-0.50	-0.04	-0.51	-0.13	0.58	0.12	-0.38	0.11	-0.09	0.02	-0.55	0.59	-0.04	1.00
